# Supplementary material for: Effect of environmental factors in reducing the prevalence of schistosomiasis in schoolchildren: An analysis of three extensive national prevalence surveys in Brazil (1950–2018)
Source: PLoS Negl Trop Dis. 2023 Jul 17;17(7):e0010804. doi: 10.1371/journal.pntd.0010804 (PMC10374055; doi:10.1371/journal.pntd.0010804)
Supplement: S1 Note — (DOCX) [file pntd.0010804.s001.docx]

S1. Supplementary Note

General and methodological characteristics of the National Helminthological Survey of Schoolchildren (IHE) (1947–1953), the Special Program for the Control of Schistosomiasis (PECE) (1975–1979), and the National Survey on the Prevalence of Schistosomiasis and Soil-transmitted Helminth Infections (INPEG) (2010–2015).

1. General and methodological aspects of national prevalence surveys

The first Brazilian national survey was carried out by researchers Pellon & Teixeira in 1950, in the National Helminthological Survey of Schoolchildren (IHE), with operationalization starting in 1947 and continuing until 1952. This survey was published in two parts, one in 1950 [1] and the second in 1953 [2]. The second national prevalence survey, the Special Program for the Control of Schistosomiasis (PECE), which included only schistosomiasis, was carried out from 1975–1979 and published in 1981 [3]. The third and last prevalence survey, the National Survey of Prevalence of Schistosomiasis and Soil-transmitted Helminth Infections (INPEG) [4], was carried out from 2010–2015 and recently published in 2018. These three national surveys produced data on the prevalence (outcome variables) of helminths for a specified year interval without annual data. This data presentation was due to the size of the territorial extension of Brazil (the largest country in South America). Thus, the considerable distances between cities prevented data collection from several municipalities simultaneously. Hence, the midpoint of the prevalence period was adopted as a reference for collecting and treating the study's explanatory variables, following the methodology usually adopted in epidemiological studies [5]. The adopted midpoints for the three national surveys, from the first to the last, were the years 1950, 1977, and 2013.

In the three surveys, the target population was students aged 7–14, and only the INPEG included students aged 15–17. The exclusion of these samples was not significant, as described in the comparative results [4]. The description of each survey's scope, sampling, and particularities can be seen below.

National Helminthological Survey of Schoolchildren (1947–1952)

The first school helminthological survey covered 16 states nationwide, totaling 1,190 sampled municipalities. According to methodological notes in the Pellon &Teixeira survey (1953), the sampling plan adopted in the first part of the study considered the municipalities with a population of more than 1,500 inhabitants distributed among 11 Brazilian states– Sergipe, Alagoas, Pernambuco, Minas Gerais, Maranhão, Piauí, Ceará, Rio Grande do Norte, Paraíba, Bahia, and Espírito Santo – in which 440,786 schoolchildren were examined.

The second stage included municipalities from five other states (Rio de Janeiro, Paraná, Santa Catarina, Goiás, and Mato Grosso), with population nuclei of more than 250 households, which corresponded to approximately 1,250 inhabitants. These municipalities represent areas or territories corresponding to municipalities, villages, districts, or regions of more than 250 households. In addition to considering the most economically important municipalities in the two stages. A total of 174,192 schoolchildren from 313 localities were examined. The reference population consisted of schoolchildren aged 7–14. The school units of the municipal center and neighboring localities were surveyed for the selection of sampled schools, and letters and memoranda were sent to teachers and school principals with explanations and details on operationalization of the survey [1,2].

The recruitment, selection, and training of personnel were conducted through the publication of public notices, written and oral exams, and admission to preparatory courses. The courses followed the thematic guidelines regarding helminths in general, laboratory materials, parasitic stool tests (parasitological research exam in feces), and records and general tabulation of results. Each establishment (group or school) received the necessary material for collection, packaging, and shipment of the sampled materials to be examined. Subsequently, the data obtained regarding *S. mansoni*, hookworms, and “helminths as a whole” were tabulated (the section “helminths as a whole” included all positive tests for any helminth). According to the record forms attached to the survey, the positivity for “helminths as a whole” was considered a multi-parasitic count per examined child, including ascariasis, trichuriasis, strongyloidiasis, teniasis, hymenolepiasis, and enterobiasis [1,2]. Spontaneous sedimentation was used in the microscopic tests following the Hoffman technique [6].

The school records accompanied by the microscopy report were sent to the heads of the survey's implementation teams, who, in turn, organized the maps of the results and submitted weekly telegrams, indicating the number of exams performed, to the Health Organization Division of Brazil’s Ministry of Health (Divisão de Organização Sanitária do Ministério da Saúde – DOS/MS). The maps and all school records (originals and two typed copies) were collected at the end of the survey and sent to the DOS/MS, along with collection material and survey reports, for verification and analysis.

It is important to note that these data are currently filed at the Instituto René Rachou/Fiocruz Minas and documented in photocopies duly typed and tabulated for the present study.

Special Program for the Control of Schistosomiasis (PECE) (1975–1979)

The Special Program for the Control of Schistosomiasis (Programa Especial de Controle da Esquistossomose – PECE) was established by Brazil’s Ministry of Health and implemented by the Ministry of Health Office of the Superintendent of Public Health Campaigns (Superintendência de Campanhas de SaúdePública – SUCAM). The PECE was a model focused mainly on conducting extensive surveys, obtaining stool test examinations, and providing and overseeing mass medical treatment of the population with oxamniquine, a drug considered to have low toxicity and easy administration at that time [7].

The attack phase of the PECE was tasked with carrying out geographical recognition and microscopic and malacological studies. Although scarce, official documents with preliminary results published during the period of the PECE indicate that the survey was carried out in municipalities representative of homogeneous microregions [8]. This territorial division was adopted from 1966 onwards to replace the 1945 division by physiographic zones. The Brazilian Institute of Geography and Statistics (IBGE) currently adopts the division by geographic meso- and microregions [9]. It is important to note that due to the very definition of concepts, PECE design objectives, and the subsequent discontinuity of the program, the final sample of the survey was of the non-probabilistic type, with municipalities in specific microregions of areas that were free, endemic, or hadvariable rates of prevalence of the disease that did not represent all geographic regions of Brazil. However, in that survey, 447,779 schoolchildren were analyzed, resulting in 30,068 positive tests, showing a prevalence of 6.7%. Initially, the survey covered only six states in Northeastern Brazil (Ceará, Rio Grande do Norte, Paraíba, Pernambuco, Alagoas, and Sergipe). Subsequently, the other regions were analyzed by including 327 municipalities from the 3,991 existing at the time (according to the 1970 Demographic Census, there were 3,952 municipalities) [10]. The Kato-Katz method was the diagnostic technique used to examine schoolchildren of the same age group included in the survey by Pellon & Teixeira [1,2].

This survey did not investigate the Federal District or the states of Acre, Amazonas, Amapá, Roraima, Rondônia, Bahia, and São Paulo. The state of Bahia was not included in this sampling because the activities of the PECE in this state began only in 1979 (after the period defined for this study) in the Paraguaçu Basin, considered an endemic area. However, the survey's conclusion in this region only occurred years later, with the completion of 482,509 stool test examinations indicating a disease prevalence of 15.68% [11]. For states in the Northern region, Pará was the only of the three states recognized by the territorial division conducted by the National Commission for Planning and Geographical-Cartographic Standards of IBGE (Amazonas, Pará, and Acre) included in the survey. The most recent and current political-administrative configuration and delimitation of the Northern Region occurred only after 1980, upon federal territories’ elevation to the category of states. These territories included Rondônia (in 1982), Amapá, and Roraima (after promulgation of the Federal Constitution of 1988), in addition to the creation of the state of Tocantins (separate from Goiás) [9].

Regarding the field team, the admission of new public employees to federal staff at the end of 1977 provided considerable advances in implementing the actions planned. Staff included supervisors, laboratory administrators, laboratory assistants, and public health assistants (guards). The endemic guards of SUCAM conducted parasitological surveys in the municipalities [12]. The PECE survey solely addressed schistosomiasis without research into soil-transmitted helminth infections, and its information was obtained through active search, including identification, diagnosis, and report of the number of patients with the disease. The reference population of the PECE was children and adolescents aged 7–14 registered in the public school system. This survey occurred in municipalities where the program had been implemented by determination of the Ministry of Health, therefore all municipalities adhered to PECE were included. The criteria for inclusion of schools and students were obtained through census surveys and an active search in school classes.

Due to the diverse methodologies adopted in implementing PECE activities in different endemic areas, several variations were adopted throughout this program in specific locations [13]. However, the basic program activities consisted of registering the localities, population, schools, and water collections that could serve as breeding sites for the intermediate host and testing the population. Like the survey by Pellon &Teixeira (1950), the PECE survey, with all its data regarding the prevalence of the disease in the sampled Brazilian municipalities, was duly typed and tabulated for the present study and documented in photocopies.

National Survey on the Prevalence of Schistosomiasis and Soil-transmitted Helminth Infections (INPEG) (2010–2015)

The National Survey on the Prevalence of Schistosomiasis and Geo helminthiasis (*Inquérito Nacional de Esquistosomose e Geo-helmintose* – INPEG) was a cross-sectional, population-based study with the reference population that consisted of children aged 7–17, attending school units in the public and private school networks of the sampled area. It was the first survey to cover all states of the Federation in an attempt to understand the prevalence of schistosomiasis, trichuriasis, hookworm, and ascariasis. Concerning comparability between the surveys, considering that the first two surveys examined only schoolchildren aged 7–14, the inclusion of schoolchildren aged 15–17 (3.68% of the total) did not statistically influence the last survey results, and for this reason they were maintained in this survey.

According to information from the survey conducted by Katz [4], the sampling plan included four epidemiological regions: an endemic area for schistosomiasis consisting of municipalities of more than 500,000 inhabitants in16 States; an endemic area consisting of municipalities of fewer than 500,00 inhabitants from 12 States; a non-endemic area for schistosomiasis consisting of municipalities of fewer than 500,000 inhabitants in 26 States; and a non-endemic area consisting of municipalities of more than 500,000 inhabitants from 14 States.

Sample size calculations were performed using previous prevalence values. For the endemic area of municipalities with less than 500,000 inhabitants, the sampling error of 5% and a power of 90% were used to determine sample size. For non-endemic and endemic areas of 500,000 inhabitants or more, the sampling error was 5%, and power was 80%. Hence, 197,564 students (89.81% of the planned participants) were examined in 521 municipalities (96.1% of the planned municipalities) [4]. The sample design included the stratification in three categories of endemic level for municipalities in the former endemic area for schistosomiasis (non-endemic area, area of low and high prevalence). Stratification of the municipalities also occurred using four population size categories (fewer than 20,000, between 20,000 and 150,000, between 150,000 and 500,000, and more than 500,000 inhabitants).

Sample selection took place through a draw among those municipalities in the sampled area, elementary schools (public and private), and between the classes in which all students were invited to provide material for the parasitological stool test. Teachers and guardians were given instructions on collection and storage procedures, and each student received an informed consent form (ICF) to be signed by parents or legal guardians. All children with positive results were referred for care in a health unit. Around 550 technicians from every state of the Federation were trained, and fieldwork was conducted by state and municipal supervisors of the Schistosomiasis Control Program (*Programa de Controle da Esquistossomose* – PCE) and municipal health agents. The method used for stool testing was the Kato-Katz parasitological diagnostic method [14], the diagnostic technique currently recommended by the World Health Organization and the PCE.

The survey form adopted was the same used in the surveys conducted by the PCE, which contains operational data input fields, even for soil-transmitted helminth infection. In this survey, the data were entered by a specialized firm hired for this purpose, using EpiData software. In addition, the research data are available for full public access on the webpage of the Department of Information Science of the Unified Health System (*Departamento de Informática do Sistema Único de Saúde* – DATASUS). National Research was approved by the Research Ethics Committee of the René Rachou Research Center – Oswaldo Cruz Foundation (Fiocruz) and by the National Research Ethics Committee (*Comissão Nacional de Ética em Pesquisa* – CONEP).

REFERENCES

1. Pellon AB, Teixeira I. Distribuição da esquistossomose mansônica no Brasil. Divisão de Organização Sanitária do Ministério da Saúde. Rio de Janeiro: MS; 1950.

2. Pellon AB, Teixeira I. O Inquérito helmintológico escolar em cinco Estados das regiões: leste, sul e centro-oeste. Divisão de Organização Sanitária do Ministério da Saúde. Rio de Janeiro: MS; 1953.

3. Brasil. Ministério da Saúde. Levantamento Nacional de Prevalência da esquisstossomose mansoni, 1975 -1979. Programa Especial de Controle da Esquistossomose. Brasília; 1981.

4. Katz N. Inquérito Nacional de Prevalência da Esquistossomose mansoni e Geo-helmintoses. Belo Horizonte: CPqRR; 2018. 76p p. (Série Esquisstossomose).

5. OPAS. Módulos de Princípios de Epidemiologia para o Controle de Enfermidades. Módulo 3: medida das condições de saúde e doença na população. Brasília, DF: Organização Pan-Americana da Saúde: Ministério da Saúde; 2010. 94 p.

6. Hoffman WA, Pons JA, Janer JL. The sedimentation concentration method in Schistosomiasis mansoni. J Publ Health and Trop Med. 1934;9(Colombia University/Puerto Rico):283–98.

7. Barbosa FS, Barbosa FS. Determination and control of schistosomiasis. Memórias do Instituto Oswaldo Cruz. 1995 Apr;90(2):155–9.

8. Brasil. Ministério da Saúde. Perspectiva do Programa Especial de Controle de Esquistossomose. [Internet]. Superintendência de Campanhas de Saúde Pública (SUCAM); 1980. Availablefrom: https://bvsms.saude.gov.br/bvs/publicacoes/0214cns_tema1_2.pdf

9. Lima M. Divisão territorial brasileira. Rio de Janeiro. Instituto Brasileiro de Geografia e Estatística (IBGE); 2002.

10. Brasil. Ministério da Saúde. Perspectiva do Programa Especial de Controle Esquistossomose Mansônica. [Internet]. SUCAM; 1981. Available from: https://bvsms.saude.gov.br/bvs/publicacoes/0214cns_tema1_2.pdf

11. Vieira JBF. O programa brasileiro de controle da esquistossomose. IV Simpósio Internacional de Esquistossomose. Rio de Janeiro; 1993.

12. Barbosa CS, Favre TC, Amaral RS, Pieri OS. Epidemiologia e controle da Esquistossomose mansoni. In: Schitosoma mansoni e esquistossomose: uma visão multidisciplinar. CARVALHO, OS., COELHO, PMZ., and LENZI, HL., orgs. Rio de Janeiro: Fiocruz; 2008. p. 964–1008. (In:; vol. 4).

13. Costa MFL e, Guerra HL, Pimenta Junior FG, Firmo JOA, Uchoa E. Avaliação do Programa de Controle da Esquistossomose (PCE/PCDEN) em municípios situados na Bacia do Rio São Francisco, Minas Gerais, Brasil. RevSocBras Med Trop. 1996 Apr;29:117–26.

14. Katz N, Chaves A, Pellegrino J. A simple device for quantitative stool thick-smear technique in Schistosomiasis mansoni. Rev Inst Med Trop Sao Paulo. 1972 Dec;14(6):397–400.
