# Supplementary material for: Effect of environmental factors in reducing the prevalence of schistosomiasis in schoolchildren: An analysis of three extensive national prevalence surveys in Brazil (1950–2018)
Source: PLoS Negl Trop Dis. 2023 Jul 17;17(7):e0010804. doi: 10.1371/journal.pntd.0010804 (PMC10374055; doi:10.1371/journal.pntd.0010804)
Supplement: S2 Note — (DOCX) [file pntd.0010804.s002.docx]

S2. Supplementary Note

**1 - Methodological criteria for inclusion and exclusion of municipalities from the study considering the process used in their establishment.**

In Brazil, from a historical point of view, the establishment of municipalities and their minimum conditions for territorial emancipation involved historical, economic, geographical, sociological, political, and legal aspects. These last two aspects are essential for the compliance industry and legal field. The emancipatory wave occurring in the country after the 1940s, linked to increasing population growth rates, promoted the establishment of a high number of municipalities, which often lacked minimum political organization or well-delimited territories [1]. According to the demographic censuses of 1940 and 1950 [2,3], Brazil had 1,574 and 1,889 municipalities, respectively, indicating the establishment of 315 new municipalities during that decade. In the following decade, 877 more municipalities were established [4].

Hence, from 1948 to 1953, the approximate date of the Pellon & Teixeira survey (1950), 168 municipalities were established [4]. However, during the intercensal periods mentioned above, many areas sampled in the survey had limited correspondence with the censuses. They were denominated as simply "localities," with no link with the territorial division adopted by the censuses, like villages (county administrative centers or suburban settlements) or cities (municipal administrative centers or urban settlements) [2]. In addition, some cities in the demographic censuses of that time lacked even a nominal description of all their villages. This situation made it difficult to officially identify the locations sampled in the survey using their corresponding information in the Demographic Census. Thus, the effects of rapid emancipation of municipalities during that period contributed to the difficulty in reorganizing the framework of political-administrative units and defining the limits of the toponymy of cities soon after the Brazilian Institute of Geography and Statistics (IBGE) was established in 1936 [5].

To carry out this ecological study, 1,152 (96.8%) of the 1,190 locations met the criterion of sufficient quality of prevalence records. Given the considerations described regarding establishment of municipalities and the emancipation process, 243 locations were excluded from the survey. This exclusion represents a loss of 26.7% for the first cross-sectional study by Pellon & Teixeira (1950). To verify the consistency of the adopted exclusion criterion, a data normality test (p-value < 0.001) was performed, indicating a non-normal distribution; then, the Wilcoxon-Mann-Whitney test (p-value = 0.1498) was performed, indicating that there was no difference between prevalence (group 1: sample of all municipalities surveyed; group 2: sample after applying the inclusion and exclusion criteria of the study). Thus, although the high percentage of loss of localities, prevalence in the smaller sample (Group 2) has a similar behavior with respect to the complete data (Group 1).

In 1970, Brazil had 3,952 municipalities and an estimated population of 4,064,183 students. For the second prevalence survey of 327 cities surveyed, promoted by the PECE [6], 30 (9.2%) did not meet the criterion of good quality records. However, of the remaining 297 cities, only 4 (1.3%) did not have compatibility with the official conformation in the demographic census at the time. All municipalities were included in the study for the third and last survey [7].

- 1. Sample characteristics after applying the inclusion and exclusion criteria.

After applying the inclusion and exclusion criteria, the remaining 1,721 municipalities in the study, per survey period, were: 907 for 1947–1953, 293 for 1975–1979, and 521 for 2010–2015. The number of repeated municipalities considering all combinations of survey periods was: 161 for 1947–1953 and 1975–1979, 57 for 1975–1979 and 2010–2015, 146 for 1947–1953 and 2010–2013, and just 41 municipalities for the three surveys together. Thus, 1,398 different municipalities are included in the study with observation in at least one of the three surveys. The survey of 1950 included 16 units in both phases, followed by the 1977 survey with 18, while 26 federal units and the Federal District were included in the survey of 2010-2015. All regions of the country were sampled in the three surveys, except for the North region in 1950, due to its not yet constituted political-administrative delimitation.

**2 - Methodological criteria and details about the techniques used for each variable and the number of observations involved for municipalities.**

To better detail the harmonization and projection process between the variables used in the three study periods, the techniques used for each variable and the number of affected observations will be described. As a first clarification, since data on the prevalence of schistosomiasis in each survey refer to annual intervals, the midpoint of each interval was adopted as a reference period for collecting and treating explanatory variables. Thus,1950, 1977 and 2013 were considered these references.

Thus, we chose to estimate the approximate year with the variable response to the study (1977 and 2013), because the information in the variable response, obtained in the Brazilian demographic censuses, did not correspond to the approximate year of the variable response. The data available for the years 1977 corresponded to the years 1970 and 1980 for the second period, and for the last period the years 1991, 2000 and 2010. We emphasize that estimation techniques are widely used, including for studies with recent data as a way to more accurately estimate the explanatory variable [8,9,10,11].

For the year 1950, among the 5442 observations that referred to 907 observations for each of the 6 explanatory variables included, only the variables "sewage network" and "Municipal GDP per capita" were obtained by means of estimates, total of 1814 observations (33.33% of the total). Projection for sanitary sewage is justified because there is no municipal information on the presence of a sewage collection network since the census of 1950 only reported the presence (or absence) of a toilet at the residence. That makes it difficult to harmonize and standardize this variable in the three analyzed periods; since information about sanitary sewage was available at the state level, the *AiBi* (or projection) was used to create such an information at the municipal level. As for municipal gross domestic product (GDP) *per capita*, the projection is justified because no information was available for the year corresponding to the 1950 reference period.

For the year 1977, all variables were estimated by linear interpolation, from the census information of the years 1970 and 1980, to obtain the closest values to the average reference year (1977). The classifications for the variable sanitary sewage (general network, septic tank, rudimentary pit and other drainage) were incorporated in the demographic census at the municipal level from the census of 1960, which allowed the direct collection of the data from 1970 and 1980 with this classification, therefore, without the need for estimates. The variable "municipal GDP per capita" was estimated by values made available in quinquennium by the IPEA, that is, by the values of the years 1970, 1975 and 1980, and its various combinations between the years, so that it was possible to obtain a better estimate of the interpolated value. Thus, all 1758 observations were estimated by linear interpolation.

For 2013, all variables were estimated using linear interpolation from 1991, 2000 and 2010 onwards for a better estimate. Then, the variables were processed using a extrapolation method to obtain the best approximation of the baseline mean (2013). For the variable "municipal GDP per capita", the best fit was polynomial extrapolation (using the Pearson correlation coefficient obtained for each adjustment). Based on annual GDP values from 1999 to 2010, values for 2011, 2012 and 2013 could be estimated through extrapolation. For the other variables, after interpolation, different extrapolation methods were performed to determine which model was closer to the real data. Thus, all observations of the year 2013 (n = 3126) were obtained by estimates [12]. Table 1 describes the variables used and the number of observations obtained by the estimation techniques, for each period as described below.

Table 1: Description of variables and observations obtained by the techniques linear and polynomial interpolation, extrapolation and Apportionment Method (*AiBi* projection).

*AiBi*: Apportionment Method. OUP: observation using projections. LIT: linear interpolation techniques. LPIE:linear and polynomial interpolation and extrapolation techniques.

**REFERENCES**

1. Ferrari S. Criação de municípios e debate científico: entre mitos e métodos. Revista de Informação Legislativa. 2016;53(211):55–80.

2. Brasil. Censo Demográfico do Brasil 1950. [Internet]. Instituto Brasileiro de Geografia e Estatística (IBGE). Série Nacional.1956; 1:265-277; 1950 [citado 10 de agosto de 2020]. Disponível em: https://biblioteca.ibge.gov.br/visualizacao/periodicos/67/cd_1950_v1_br.pdf

3. Brasil. Censo Demográfico do Brasil 1940. [Internet]. Instituto Brasileiro de Geografia e Estatística (IBGE). Série Nacional.1956; 1:265-277; [citado 10 de agosto de 2020]. Disponível em: https://biblioteca.ibge.gov.br/visualizacao/monografias/GEBIS%20-%20RJ/CD1940/Censo%20Demografico%201940%20VII_Brasil.pdf

4. Furtado BA. EVOLUÇÃO DA DIVISÃO TERRITORIAL DE MINAS GERAIS: OS LIMITES MUNICIPAIS DESDE 1711. GEOGRAFIA. 2007;32(1):199–213.

5. Vilarinho PF. A trajetória do aprendizado tecnológico nos censos demográficos no Brasil. Cadernos EBAPEBR. 1^o^ de janeiro de 2006;4(2):1 a 17–1 17.

6. Brasil. Ministério da Saúde. Levantamento Nacional de Prevalência da esquisstossomose mansoni, 1975 -1979. Programa Especial de Controle da Esquistossomose. Brasília; 1981.

7. Katz N. Inquérito Nacional de Prevalência da Esquistossomose mansoni e Geo-helmintoses. Belo Horizonte: CPqRR; 2018. 76p p. (Série Esquisstossomose).

8 Xia Q, Maduro GA, Li W, Huynh M, Torian LV. Life Expectancy Among People With HIV in New York City, 2009-2018. J Acquir Immune Defic Syndr. 2022;91: 434–438. doi:10.1097/QAI.0000000000003095

9. Markley SN, Holloway SR, Hafley TJ, Hauer ME. Housing unit and urbanization estimates for the continental U.S. in consistent tract boundaries, 1940-2019. Sci Data. 2022;9: 82. doi:10.1038/s41597-022-01184-x

10. Souza AA de, Mingoti SA, Paes-Sousa R, Heller L. Combined effects of conditional cash transfer program and environmental health interventions on diarrhea and malnutrition morbidity in children less than five years of age in Brazil, 2006–2016. PLOS ONE. 2021;16: e0248676. doi:10.1371/journal.pone.0248676

1. Poague KIHM, Mingoti SA, Heller L. Water, sanitation and schistosomiasis mansoni: a study based on the Brazilian National Prevalence Survey (2011 – 2015). Ciênc saúde coletiva. 2022. Available: https://cienciaesaudecoletiva.com.br/artigos/water-sanitation-and-schistosomiasis-mansoni-a-study-based-on-the-brazilian-national-prevalence-survey-2011-2015/18447?id=18447
2. Santos J, Gibim G. Cálculo numérico. In: Unidade 3: interpolação. Vol. 1. Londrina: Editora e Distribuidora Educacional S.A; 2015. 216 p.
