# Supplementary material for: Effect of environmental factors in reducing the prevalence of schistosomiasis in schoolchildren: An analysis of three extensive national prevalence surveys in Brazil (1950–2018)
Source: PLoS Negl Trop Dis. 2023 Jul 17;17(7):e0010804. doi: 10.1371/journal.pntd.0010804 (PMC10374055; doi:10.1371/journal.pntd.0010804)
Supplement: S3 Note — (DOCX) [file pntd.0010804.s003.docx]

S3. Supplementary Statistical Analysis

1. **Models Definition**

The main response variable considered in the statistical analysis is a count variable. For each of the 1,721 municipalities the number of positive results for Schistosomiasis among children sampled from the municipality is available as well as the respective prevalence, which is defined as the ratio between the “Number of students with positive stool tests” and the “Total number of students aged 7-14 examined in the respective municipality”.

In the statistical model the Poisson and Negative Binomial distributions were used to describe the probabilistic pattern of the count variable for Schistosomiasis and the respective number of sampled children was the exposure variable (offset) [1,2]. The link function was the logarithm of the mean (the Poisson and Negative Binomial distributions are part of the exponential family). Therefore, with this modeling it is possible to estimate prevalence as well as the number of positive cases for each municipality, including explanatory (independent) variables to explain the response mean.

Models with the adjustment for excess of zero were employed in order to verify the robustness and consistency of the analyses (municipalities with zero cases 45.4% for 1947-1953 period; 54.2% for 1974-1979 period and 73.7% for 2010-2015 period). In this approach it is possible to adjust a regression model which considers the frequency of zero generated by the Poisson (and Negative Binomial) as well as the frequency of zero that is generated by another distribution related to the excess of zero [3,4]. Although there are many distributions that can be used with this purpose, the logistic distribution is very common. In the adjustment of zero inflated models, two probability distributions (Poisson (or Negative Binomial) and Logistic) are combined to generate estimates of the number of cases and prevalence for each sample unit (municipality) considering the respective values of the explanatory variables included in the models.

Poisson and Negative Binomial Generalized Linear Mixed Models (GLMMs) [5,6,7] with random effects related to three levels (Level 1: region; Level 2: state; and Level 3: municipality) were applied, with and without zero-inflation. These three hierarchical levels of data were incorporated into the random intercepts of the GLMMs to allow the joint modeling of data from the different municipalities of each sampling period. That is, the option of using GLMMs is based on the fact that it allowed to use the information from all municipalities of the three surveys (n=1721) in the analysis and improving the estimation of the parameters of the model, respective standard deviations and p-values. It is well known that multilevel models (with random effects) provide better inference from grouped data (in the case of the presented study, students are grouped in municipalities which are grouped in states which are grouped in regions) since the coefficient and variance error for each explanatory variable are better estimated, avoiding the problem of underestimation of coefficients and overstatement of their significance that occur when clustering effect is not taken into account [5,6].

For each quantitative independent variable, the RR presents the ratio between the average prevalence of positive cases of the Schistosomiasis considering a situation of an increase of 1 unit of measurement in the respective independent variable. Thus, it is an estimate of the change of the average prevalence as the independent variable increases by an additional unit, being the calculation performed considering that all the other independent variables included in the model are fixed. The maximum likelihood statistical procedure was used to estimate the parameters of the models in software R using package *glmmTMB* [7].

Four GLMMs were fitted to the schistosomiasis data: Poisson and Negative Binomial, with and without excess of zero. The following selection criteria were considered in definition of the final regression model: firstly, Poisson or Negative Binomial distribution were compared according to the lowest residual variance. Then, consideration of zero inflation was defined with basis on the lowest values for the Akaike Information Criterion (AIC) and the Bayesian Information Criterion (BIC) [8]. Results are shown in Table S3.1. It shows that the residual variance from both Poisson models (with and without inflation) is smaller than for both Negative Binomial models. Among the Poisson models the zero-inflated model had lower AIC and BIC values. Table S3.1 complements the results presented in Table 4 of the paper. The use of zero-inflation model makes sense since the schistosomiasis data showed a large number of zero cases in all 3 surveys, especially for the period 2010-2015 (45.4% for 1950; 54,2% for 1977; 73.7% for 2013).

**Table A:** **Goodness-of-fit measures of multilevel** **Poisson and Negative Binomial models, with and without zero inflation, fitted to schistosomiasis data**

| **Model** | **Residuals Variance** | **AIC** | **BIC** |
| --- | --- | --- | --- |
| Poisson | 520.87 | 12,861.4 | 12,926.8 |
| Zero-inflated Poisson | 4,283.5 | 11,162.2 | 11,233.1 |
| Negative Binomial | 10,745.6 | 8,322.2 | 8,387.6 |
| Zero-inflated Negative Binomial | 10,843.2 | 8,324.2 | 8,395.1 |

Akaike Information Criterion (AIC); Bayesian Information Criterion (BIC)

1. **Predictive Analysis**

Table S3.2 displays some summary statistics for the observed and predicted schistosomiasis prevalence in each period. It can be seen that the fitted statistical model predicted the same decreasing behavior among the three sampling surveys as observed in the data. Therefore, in general, the model provided similar and consistent results with those observed in the data, corroborating its adequacy.

Table B: Descriptive statistics on the prevalence of schistosomiasis per study period in the 1,721 sampled Brazilian municipalities: Observed and predicted values using the zero-inflated Poisson multilevel regression model.


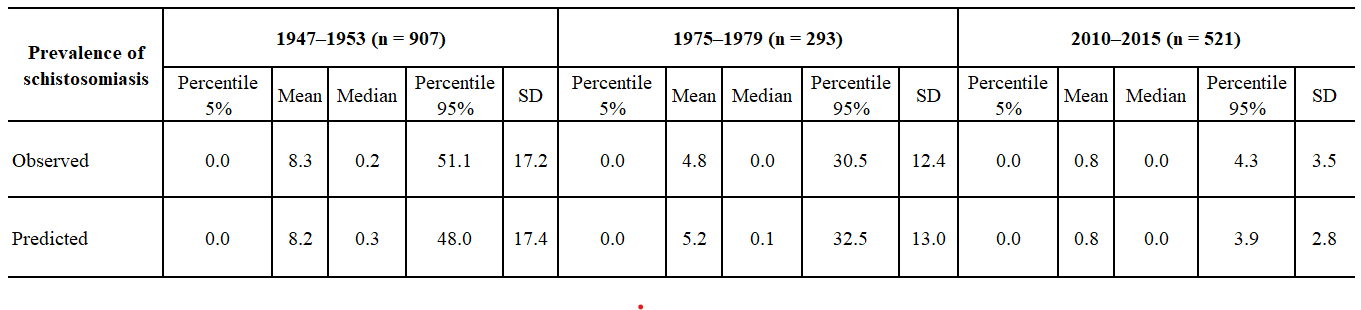

SD: Standard Deviation. n=number of sampled municipalities.

1. **Sensitivity Analysis**

Statistical models were fitted using the data set composed of 41 common municipalities among the three surveys. In this case, as there are no repeated municipality in the data, we considered usual generalized linear models (GLM) using Poisson and Negative Binomial distributions, with and without excess of zero. The model with best fit for the same set of explanatory variables included in the multilevel model presented in Table 4 of the manuscript was also the Poisson with zero-inflation. As shown in Table S3.3, the coefficients and RR estimates are similar to results obtained by the multilevel model, which consider the data of all municipalities in all periods. Modeling of zero-inflation probabilities included more explanatory variables in this case, however keeping the %Urbanization with a similar effect to the multilevel model. The model effect comparing years 1950 and 1977 was not significant but prevalence decreased from 1950 to 2013. Therefore, in general the results are consistent with the multilevel model presented in the manuscript (Table 4). It is important to point out that the results of the multilevel model are more precise since a larger number of municipalities were used in the estimation of the parameters of the models.

**Table C: Results from the zero-inflated Poisson regression model fitted to assess the prevalence of schistosomiasis mansoni in the 41 common Brazilian municipalities among the three surveys.**

| **Coefficient** | **Poisson regression** | | | | |
| --- | --- | --- | --- | --- | --- |
|  | **RR** | **(CI 95%)** | **Estimate** | **P-value** |  |
| Model constant (intercept) | - | - | -0.070 | 0.589 | |
| LN Population | 0.887 | (0.867 – 0.908) | -0.120 | <0.001 | |
| % Urbanization | 0.979 | (0.977 – 0.981) | -0,021 | <0.001 | |
| % Occupancy condition of the household | 0.994 | (0.993 – 0.996) | -0.006 | <0.001 | |
| % Water supply | 1.035 | (1.033 – 1.036) | 0.034 | <0.001 | |
| % Sewerage | 0.969 | (0.968 – 0.971) | -0.031 | <0.001 | |
| Year: 1977 | 1.014 | (0.966 – 1.065) | 0.014 | 0.572 | |
| Year: 2013 | 0.215 | (0.191 – 0.241) | -1.538 | <0.001 | |
| **Coefficient** | **Zero-inflation logistic regression** | | | | |
|  | **RR** | **(CI 95%)** | **Estimate** | **P-value** | |
| Model constant (intercept) | - | - | 10.203 | <0.001 | |
| LN Population | 0.522 | (0.288 – 0.755) | -0.762 | 0.002 | |
| % Urbanization | 0.904 | (0.868 – 0.940) | -0.102 | <0.001 | |
| % literacy rate | 1.007 | (1.051 – 1.162) | -0.099 | <0.001 | |
| Municipal GDP per capita | 1.403 | (1.133 – 1.672) | 0.312 | 0.001 | |
| Reference year: 1950. CI: Confidence interval. LN: natural logarithm. RR: Rate Ratio. OR: odds ratio. | | | | | |

1. **Comparison of Endemicity Level Distribution Between the Three Surveys**

As mentioned in the manuscript, the survey from 2010–2015 is the only with more published details about the sampling procedure used to collect the data. In such survey, the stratified sampling procedure was used considering size of population and a degree of endemicity for Schistosomiasis (in three different levels: non-endemic, low-prevalence and high-prevalence). Endemicity classification is not available for 1950 nor 1977.

Although the classification (or definition) of degree of endemicity may have changed from 1947-1953 to 2010-2015, the distribution of the endemic levels from 2010-2015 survey was compared with the respective distributions from 1947-1953 and 1975-1979, using the following procedure. First, the municipalities that were sampled in both 1947-1953 and 2010-2015 periods (same municipalities) were identified and associated with the respective endemic classification (Non-endemic, Low-prevalence, High-prevalence) according to the information available in the 2010-2015 period. Then, the number and proportion (percentage) of municipalities at each endemic level were calculated. Finally, the distributions of proportions observed in 1947-1953 and 2010-2015 were compared using Pearson's Chi-square statistical test. The same procedure was carried out for the 1975-1979 data.

As shown in Table S3.4, the p-values of the tests indicated that the distributions were similar (p-value=0.183 for comparison between 1975-1979 and 2010-2015; and p-value=0.598 for comparison between 1947-1953 and 2010-2015). Considering these results, it can be concluded that, although there are differences in the sampling procedures, the three samples are comparable in terms of the proportion of municipalities within each endemic level with basis on the endemicities reported in 2010-2015 survey.

**Table D: Distribution of endemic levels between surveys**

| **Period** | **Endemicity Level** | | | **P-value** |
| --- | --- | --- | --- | --- |
|  | **Non-endemic n (%)** | **Low-prevalence n (%)** | **High-prevalence n (%)** |  |
| 2010-2015 | 372 (71.4%) | 85 (16.3%) | 64 (12.3%) | - |
| 1975-1979 | 34 (56,6%) | 13 (22,8%) | 10 (17,6%) | 0.183 |
| 1947-1953 | 98 (67.1%) | 28 (19.2%) | 20 (13.7%) | 0.598 |

P-value: Pearson's Chi-squared statistical test comparing the period with 2013. n: Total number of municipalities.

**REFERENCES**

1. Dupuy,JF. Statistical Methods for Overdispersed Count Data.1o.ed. Oxford, EUA: ISTE Press Ltd and Elsevier Ltd, 2018. 180 p. https://doi.org/10.1016/C2017-0-00831-5

2. Hilbe JM. Negative Binomial Regression. 2o ed. New York, EUA: Cambridge University Press; 2011. 576 p.

3. Lambert D. Zero-Inflated Poisson Regression, with an Application to Defects in Manufacturing. Technometrics. 1992;34: 1–14. doi:10.2307/1269547

4. Greene WH. Accounting for Excess Zeros and Sample Selection in Poisson and Negative Binomial Regression Models. Rochester, NY; 1994. Available: https://papers.ssrn.com/abstract=1293115

5.B.M.Bolker. Linear and Generalized Linear Mixed Models. In G.A. Fox,S. Negrete-Yankelevich, and V.J. Sosa, editors, Ecological Statistics. Oxford University Press, Oxford, UK,2015. [p378,379]

6.B.M. Bolker, M.E. Brooks, C.J. Clark, S.W. Geange, J.R. Poulsen, M.H.H. Stevens, J.S.S. White

Generalized linear mixed models: a practical guide for ecology and evolution Trends Ecol. Evol., 24 (2009), pp. 127-135

7. Brooks, M. E., Kristensen, K., van Benthem, K. J., Magnusson, A., Berg, C.W., Nielsen, A., Skaug, H. J., Mächler, M. and Bolker, B. M. (2017). glmmTMB balances speed and flexibility among packages for zero-inflated generalized linear mixed modeling. The R Journal, 9(2), 378–400.

8. A. Chakrabarti, J.K. Ghosh

AIC, BIC and Recent Advances in Model Selection

P.S. Bandyopadhyay, M.R. Forster (Eds.), Handbook of the Philosophy of Science, Philosophy of Statistics, Elsevier, Amsterdam, The Netherlands (2011), pp. 583-605
