# Supplementary material for: Effect of environmental factors in reducing the prevalence of schistosomiasis in schoolchildren: An analysis of three extensive national prevalence surveys in Brazil (1950–2018)
Source: PLoS Negl Trop Dis. 2023 Jul 17;17(7):e0010804. doi: 10.1371/journal.pntd.0010804 (PMC10374055; doi:10.1371/journal.pntd.0010804)
Supplement: Attachment — Submitted filename: PLOS Neglected Tropical Diseases-09-22.docx (1).pdf [file pntd.0010804.s004.pdf]

PLOS Neglected Tropical Diseases

Article:

Effect of environmental factors in reducing the prevalence of schistosomiasis in schoolchildren: A panel analysis of three extensive national prevalence surveys in Brazil (1950–2018)

1. **Summary of strengths, weaknesses, and overall contribution:**
   a. This is an ecological study that analyzes three different Brazilian Schistosomiasis surveys and proposes a series of factors that could explain a reduction in the prevalence of this disease among schoolchildren between 1950-2018.
   b. The authors design a robust statistical inference framework and declare that, although there are many differences in the surveys (methods for diagnosis and sampling, for example), "the findings of this study indicate a decrease in the prevalence of schistosomiasis over seven decades in schoolchildren from the analyzed Brazilian municipalities, mediated by environmental factors and social conditions."
   c. This paper could contribute to the implementation of public policies, like larger access for sanitization, planned urbanization, and a proposition for a long-term policy for household ownership.
   d. The main problem of this paper is methodological: the bias for the lack of "harmonization between variables to allow comparisons"; the fact that only 41 municipalities repeated the three surveys together; the diagnosis method is different in the first survey from the other ones; the second survey "consisted of a non-probabilistic sample". This problem compromises all the findings of this paper.

1. **Major comments:**
   a. Study design (109): the authors suggest that three cross-sectional studies analyzed together consist of a longitudinal study. Please, explain better or review the term "longitudinal" for the study design.
   b. (149-151): the authors state the superiority of the Kato-Katz method, but in the first survey, spontaneous sedimentation was used. Please, explain how the authors standardized the prevalence with such different methods for

Schistosomiasis diagnosis.

c. Inclusion and exclusion criteria (170-193): Only 41 municipalities are repeated for the three surveys, and the authors chose 1,721 municipalities to analyze. Please, explain how it was possible to infer the prevalence fall with this data, and how to infer the independent variables, if it was selected from the first survey 907 municipalities vs 293 and 521 municipalities from the second and third surveys, respectively.
d. Please, clarify the affirmation of the findings of this research in lines 488-490.

1. **Minor comments:**
   a. Table 1: Municipal GDP per capita - please correct PECE period “975”.
   b. We suggest expanding the introduction section with some elements of the discussion, like the factors related to the availability of piped water (422, 434) or the increased risk related to rented houses “compared with families who owned their housing” (456-459).
   c. We could not find the reference for Grimes et al (2014) - line 500.
   d. We suggest reviewing line 505. If this study is not generalizable, then how could affirm that this study indicates a decrease in the prevalence of schistosomiasis over seven decades from the analyzed municipalities?

1. **Limitations:**
   a. The method, as described above, is the main limitation of the current paper. The authors should carefully review and better explain how to solve the bias.

1. **Supplementary Materials:**
   a. none to declare.
   b. The tables and pictures are adequate and well explored.

1. **Title and abstract:**
   a. Title: none to declare;
   b. abstract: please review the recommendations and, if possible, some of the suggestions.
