# Supplementary material for: Effect of environmental factors in reducing the prevalence of schistosomiasis in schoolchildren: An analysis of three extensive national prevalence surveys in Brazil (1950–2018)
Source: PLoS Negl Trop Dis. 2023 Jul 17;17(7):e0010804. doi: 10.1371/journal.pntd.0010804 (PMC10374055; doi:10.1371/journal.pntd.0010804)
Supplement: Attachment — Submitted filename: Comments1.pdf [file pntd.0010804.s005.pdf]

Review Report

The manuscript "**Effect of environmental factors in reducing the prevalence of schistosomiasis in schoolchildren: A panel analysis of three extensive national prevalence surveys in Brazil (1950–2018)**" presents an interesting analysis of schistosomiasis over seven decades in schoolchildren in **Brazil**. This study will provide useful information to those working in environmental and healthcare delivery in **Brazil**. Please find the following specific comments:

The language should be checked, and some parts of the paper may require additional work or clarification.
A map of Brazil showing the administrative divisions (regions) and/or a description of how the administrative regions differ from states (level 2) and municipalities (if possible) would be helpful in explaining some of the differences in prevalence (as you mentioned in the methodology that the municipality as the unit of analysis).

Methods/Methodology

What are the motivating explanations for the use of Zero-inflated Poisson regression models in this manuscript?

Poisson and Negative Binomial regressions, with and without zero inflation in both cases, are used; however, no explanation of the models is given. A clearer explanation of the models would be helpful in understanding the results from each.

The authors mentioned that the outcome variable was the prevalence of schistosomiasis in schoolchildren from seven to 14 years old, which means the Infection status (positive/negative) for schistosomiasis in schoolchildren from seven to 14 years old. How?? (using Poisson with zero inflation)

Data source

"This survey also considered schoolchildren aged 7-14 by using a cluster sampling plan...", line 136. There is a contradiction in the explanation given in line 155, which specifies "....also included students aged 15-17."

Data analysis

A backward selection procedure was used to identify the significant fixed effects, considering a 25% significance level for the removal of an explanatory variable. Why the authors considered 25%?

Results

1- The results of the fitted zero-inflated Poisson regression model for the various periods are not shown in the manuscript.
2- The results of the Negative Binomial regression model for the various periods are not shown in the manuscript.

3-The criteria values (residual variance, AIC, and BIC) generated by the different models should be presented in the manuscript.
4-It is critical to use the same number of decimal places for all values in the manuscript. For example, the authors used two decimal places in table 3 but only one in the explanations.
5-Line 286, the value described is not correct [758 (44.0%)]. It should be 788 (45.78%).
6-Line 288, the Northeast and Southeast regions had the highest percentages of municipalities in each survey. This statement should be rewritten as “The Northeast region had the highest percentage of municipalities in each survey following by the southeast……”
7-The percentage of the subtotal in table 3 for the Northeast region from 1947 to 1952 is incorrect, also the number of municipalities from 1975 to 1979 (114 vs 144).
8-The naming of the regions is not consistent. For example, Central-West and Midwest.
9-The results are not well explained.

Discussion

The results are not well discussed.

References

The references are not organized at all.

Decision

The paper can .be published upon addressing the above comments.
