# Supplementary material for: Effect of environmental factors in reducing the prevalence of schistosomiasis in schoolchildren: An analysis of three extensive national prevalence surveys in Brazil (1950–2018)
Source: PLoS Negl Trop Dis. 2023 Jul 17;17(7):e0010804. doi: 10.1371/journal.pntd.0010804 (PMC10374055; doi:10.1371/journal.pntd.0010804)
Supplement: Attachment — Submitted filename: Letter to the Review Editor PNTD final.docx [file pntd.0010804.s006.docx]

**LETTER TO REVIEWERS**

Dear Editor,

We thank the reviewers for the valuable observations and suggestions for improving our manuscript, which have greatly contributed forthe quality of this new version. We tried to accommodate most of the suggestions, certain of their relevance and appropriateness. The changes and additions made are highlighted in the text file sent and in this letter. When the comments were not clearly described in our view, we addressed them based on our understanding. Please find below a point-by-pointcommnents.

**Reviewer 1**

- *Study design (109): the authors suggest that three cross-sectional studies analyzed together consist of a longitudinal study. Please, explain better or review the term “longitudinal” for the study design.*

Reply: changed sentence “The epidemiological design of the research consists of a longitudinal study on three panels containing observational ecological data” to “The epidemiological design of the research consists of a prospective study, covering three panels with observational ecological data”. (lines 110-111)

- *(149-151): the authors state the superiority of the Kato-Katz method, but in the first survey, spontaneous sedimentation was used. Please, explain how the authors standardized the prevalence with such different methods for Schistosomiasis diagnosis*.

Reply: changed to “Regarding the parasitological tests used in the surveys, although two different methods were used, the comparability between them is possible. Firstly, during the 1947-1952 IHE, Brazil had a high prevalence of schistosomiasis and a high intensity of infection, implying that the application of less sensitive diagnostic methods, such as the technique of spontaneous sedimentation in water (Hoffman, Pons et al. Janer, or HPJ technique) [69], leads to a low number of false-negative [70]. Secondly, the results obtained in the two last surveys (PECE and INPEG), which used the Kato-Katz method, could identify a greater number of true positive, with a low detection of false-negatives, due to superior sensitivity of the method. It is well known that the Kato-Katz method is currently the gold standard method recommended by the WHO [3,71]”. This explanation was moved from “Methods” for “Discussion” (Lines 525-534). Two articles were included in the citations to support the authors' justification. We emphasize that, in 1950, have used less sensitive test, as HPJ, could detect low number of false-negative, since Brazil had a high prevalence and intensity infection in that period.

- *Inclusion and exclusion criteria (170-193): Only 41 municipalities are repeated for the three surveys, and the authors chose 1,721 municipalities to analyze. Please, explain how it was possible to infer the prevalence fall with this data, and how to infer the independent variables, if it was selected from the first survey 907 municipalities vs 293 and 521 municipalities from the second and third surveys, respectively.*

Reply: the inclusion and exclusion criteria allowed 1,721 municipalities to be included in the survey, so that data on prevalence and independent variables could be collected for each one of them. In the statistical analysis, the same 1,721 municipalities were controlled for their random effects and hierarchically analyzed in terms of state and region to which they belonged. The multilevel model allows joint evaluation of different municipalities and periods, controlling, through random effects, their regional, state and municipal differences, since disease control policies are similar at the regional level, especially in endemic locations.

The multilevel model provides better inference from grouped data. By considering a multilevel model, it was possible to use the information from all municipalities of the 3 surveys (n=1721) in the analysis, improving estimation of the coefficients of the independent variables as well as their respective standard errors and p-values. It is well known that multilevel models (with random effects) provide better inference from grouped data (in the case of the presented study, students are grouped in municipalities which are grouped in states which are grouped in regions) since the coefficient and variance error for each explanatory variable are better estimated, avoiding the problem of underestimation of coefficients and over statement of their significance that occur when clustering effect is not taken into account. The explanation is inserted in lines 344-356 and 535-546. For better clarification, a sensitivity analysis was performed, as described below.

- *Please, clarify the affirmation of the findings of this research in lines 488-490*.

Reply: the phrase emphasizes that different forms of access and exposure to water and sewage contribute to variations in disease infection rates over time and in various regions. The sentence has been rewritten for clarification (lines 559-562). “In line with findings of other studies, differences between forms of access and exposure to water and sanitation relate to variations in disease infection rates over time and in different regions , suggesting that the impact of access to water and sanitation is mediated by other social, behavioral, and environmental factors [73]”.

- *Table 1: Municipal GDP per capita - please correct PECE period “975”. We could not find the reference for Grimes et al (2014).*

Reply: period revised, thanks. Inserted reference in lines 562: Grimes JET, Croll D, Harrison WE, Utzinger J, Freeman MC, Templeton MR. The Relationship between Water, Sanitation and Schistosomiasis: A Systematic Review and Meta-analysis. PLoSNeglTropDis [Internet]. 4 de dezembro de 2014;8(12). Available: <https://www.ncbi.nlm.nih.gov/pmc/articles/PMC4256273/>

- *We suggest expanding the introduction section with some elements of the discussion, like the factors related to the availability of piped water (422, 434) or the increased risk related to rented houses “compared with families who owned their housing” (456-459).*

Reply:we have inserted elements related to water and sanitation in the Introduction (we felt less confident stating associations of the disease with household status in this very early section). Text in lines 72-75 was modified to: “Among them, access to safe and continuous water and improved sanitary facilities that allow for better conditions dwelling can have an important role in breaking the disease cycle, interrupting the release of eggs in the environment and avoiding access to surface water for water supply”.

- *This is an ecological study that analyzes three different Brazilian Schistosomiasis surveys and proposes a series of factors that could explain a reduction in the prevalence of this disease among schoolchildren between 1950-2018. The authors design a robust statistical inference framework and declare that, although there are many differences in the surveys (methods for diagnosis and sampling, for example), “the findings of this study indicate a decrease in the* *prevalence of schistosomiasis over seven decades in schoolchildren from the analyzed Brazilian municipalities, mediated by environmental factors and social conditions.” This paper could contribute to the implementation of public policies, like larger access for sanitization, planned urbanization, and a proposition for a long-term policy for household ownership. The main problem of this paper is methodological: the bias for the lack of “harmonization between variables to allow comparisons”; the fact that only 41 municipalities repeated the three surveys together; the diagnosis method is different in the first survey from the other ones; the second survey “consisted of a non-probabilistic sample”. This problem compromises all the findings of this paper*.

Reply: the authors are grateful for the contribution, and we acknowledge that there are limitations in our study. However, we support that analysing three large surveys, covering such a large time span with large samples is an opportunity for exploring the data and extracting possible findings of the association between environmental conditions and disease prevalence. Regarding the methodological issues raised by the reviewer, we consider that, although possible limitations, they do not compromise significantly our findings. We discussed the three first issues (the lack of harmonization between variables; the fact that only 41 municipalities repeated the three surveys; a different diagnosis method in the first survey) in our previous responses and in the manuscript.

As for the sample of the second survey, it consisted of a sample from endemic and non-endemic areas for the disease, with criteria for defining schoolchildren, schools, and municipalities such as the 1947-1952 survey. We believe that, even the sample being not probabilistic, a robust criteria for surveying and collecting data was applied, not compromising the estimates of disease prevalence. For clarification, we included an explanationin the methodology (lines 134-138): “This survey took place in municipalities where the program had been implemented by the Ministry of Health and included all municipalities that adhered to PECE. The criteria for inclusion of schools and students were based on the decennial census and an active search in school classes [13,14]”.Two references were included to support the justification.

Regarding harmonization between variables, we added information to the main text that refers to the descriptive table (item 2 Supplementary Material S2 and inserted in lines 228-230 in the manuscript) about the techniques used and the number of observations. We emphasize that all the peculiarities were not disregarded and the techniques are widely used, including for studies with more recent data as a way of more accurately estimating the variable for the year of the study.

In addition, a sensitivity analysis was performed and the resuls were introduced in the Supplementaty File S3 - Additional Statistical Analysis.

Statistical models were adjusted using the data set composed of 41 common municipalities among the three surveys.The model with best fit was also the Poisson with zero-inflation and composed of the same explanatory variables as in the multilevel model presented in Table 4 of the manuscript. The estimates of the coefficients and Rate Ratio (RR) are similar and, therefore, the results are consistent with those found in the multilevel model presented in the paper. As an advantage, the multilevel model allows evaluating different municipalities and periods, controlling,through random effects, their regional, state and municipal differences, since disease control policies are similar at the regional level, especially in endemic locations. The multilevel model provides better inference from grouped data.

Also, it was included the results of a statistical analysis comparing the distribution of the municipalities of the three surveys according to the endemicity classification used in the sampling procedure of the third survey (2010-2015). The results indicated that, although there are differences in the form of data collection regarding the selection of municipalities, the three samples are comparable in terms of the endemicity degree criterion used in the 2010-2015 survey. The results and details of these analyses were included in the Supplementary File S3. The explanation was inserted in lines 344-356 of the revised manuscript.

**Reviewer 2**

- *Yes, there are concerns about ethical or regulatory requirements.*

Reply: The study is based on secondary data, witch does not allow identification of participants. To answer this concern, the ethics statement section has been moved to the “Data Analysis” section to emphasize this information. (lines 276-281).

- *A map of Brazil showing the administrative divisions (regions) and/or a description of how the administrative regions differ from states (level 2) and municipalities (if possible) would be helpful in explaining some of the differences in prevalence (as you mentioned in the methodology that the municipality as the unit of analysis).*

Reply: We appreciate the suggestion and have inserted a description of how the administrative regions differ from states and how they were considered in analysis (lines 242-253). “According to the official territorial division proposed by the IBGE, the Brazilian political-administrative organization is divided into five macro-regions, which include 26 federal units (states) plus the Federal District and 5,570 municipalities. In order to consider this hierarchical characteristic of the data, we applied Generalized Linear Mixed Models (GLMMs) with random effects related to three levels: regions (Level 1,with 5 categories South, Southeast, North, Northeast and Midwest); states (Level 2, representing the 26 federative units plus the Federal District); and municipalities (Level 3, related to the 1,398 different municipalities included in the study with observation in at least one of the three surveys). These three hierarchical levels of data were incorporated into the random intercepts of the GLMMs to allow the joint modeling of data from the different municipalities of each sampling period”.

- *What are the motivating explanations for the use of Zero-inflated Poisson regression models in this manuscript? Poisson and Negative Binomial regressions, with and without zero inflation in both cases, are used; however, no explanation of the models is given. A clearer explanation of the models would be helpful in understanding the results from each.*

Reply: In the statistical model the Poisson and Negative Binomial distributions were used to describe the probabilistic pattern of the count variable for schistosomiasis and the respective number of sampled children was the exposure variable (offset). The link function was the logarithm of the mean (the Poisson and Negative Binomial distributions are part of the exponential family). Because of the larger amount of municipalities with zero cases of schistosomiasis (45.4% for 1947-1953 period; 54.2% for 1974-1979 period and 73.7% for 2010-2015 period), models with the adjustment for excess of zeros were employed in order to verify the robustness and consistency of the analyses. This explanation was insert in lines 11-13, 290-291 and 329-332.

In this approach it is possible to adjust a regression model which considers the frequency of zero generated by the Poisson (and Negative Binomial) as well as the frequency of zero that is generated by another distribution related to the excess of zero. Although there are many distributions that can be used with this purpose, the logistic distribution is very common. In the adjustment of zero inflated models, two probability distributions (Poisson (or Negative Binomial) and Logistic) are combined to generate estimates of the number of cases and prevalences for each sample unit (municipality) considering the respective values of the explanatory variables included in the models.

We included the discussion above in the Supplementary File S3 and also a comment in lines 258-261, 329-341 and 365-366 of the revised manuscript. Table 4 was modified to include results on the zero-inflation part of the model.

- *The authors mentioned that the outcome variable was the prevalence of schistosomiasis in schoolchildren from seven to 14 years old, which means the Infection status (positive/negative) for schistosomiasis in schoolchildren from seven to 14 years old. How?? (using Poisson with zero inflation).*

Reply: The main outcome variable is a count variable. For each of the 1,721 municipalities the number of positive results for schistosomiasis among children sampled from the municipality is available as well as the respective prevalence, which is defined as the ratio between the “Number of students with positive stool tests” and the “Total number of students aged 7-14 examined in the respective municipality”.

As explained in the answer of the previous question, in the statistical model the Poisson and Negative Binomial distributions were used to describe the probabilistic pattern of the count variable for schistosomiasis and the respective number of sampled children was the exposure variable (offset). The link function was the logarithm of the mean (the Poisson and Negative Binomial distributions are part of the exponential family). Therefore, with this modeling it is possible to estimate prevalences as well as the number of positive cases for each municipality, including explanatory (independent) variables to explain the response mean.

For each quantitative independent variable, the RR presents the ratio between the average prevalences of positive cases of the Schistosomiasis considering a situation of an increase of 1 unit of measurement in the respective independent variable. This explanation was inserted in lines 258-261 and 360-364.

- *"This survey also considered schoolchildren aged 7-14 by using a cluster sampling plan...", line 136. There is a contradiction in the explanation given in line 155, which specifies "....also included students aged 15-17.*

Reply: We have adjusted the text to make it clearer regarding differences in age groups. We emphasized that the inclusion of age groups from 15 to 17 did not result in a statistically significant difference in the sample, according to the description of the methodology and detailed results of INPEG (KATZ, 2018), and for this reason they were maintained in this survey. We believe it is pertinent to remove this information to contribute to the clarity of the text and better understanding of the methods, but in supplementary material S1 also contain this explanation.

Katz N. Inquérito Nacional de Prevalência da Esquistossomose mansoni e Geo-helmintoses. Belo Horizonte: CPqRR; 2018. 31-37p. (Série Esquisstossomose). Available in: <http://tabnet.datasus.gov.br/cgi/sinan/inpeg/RelatorioINPEG.pdf>

- *Not correct statistical analysis used to support conclusions.*

Reply: Based on comments from reviewers of this manuscript, the data analysis in section “Methods” has been improved considerably (line 242-253, 256-261, 262-269 and supplementary statistics analysis). The authors agree that there were limiting conditions and that they were carefully considered in the analyses. We also agree that the changes made based on reviewers’ comments were important contributions to improving these points. We hope that the adherence of the statistical analysis with the research question is now adequately demonstrated.

- *A backward selection procedure was used to identify the significant fixed effects, considering a 25% significance level for the removal of an explanatory variable. Why the authors considered 25%?*

Reply: Setting a higher significance level is strongly recommended in backward selection in order to allow more variables to be included in the model. Usual cut-off significance level of only 0.05 can sometimes miss important variables in the process of variable selection. After removing variables using this higher cut-off, one generally proceeds to a more detailed analysis that may possibly indicate the removal of new variables. In our study, after the first stage, all remaining variables were already significant at 5%. We rewrote the sentence appearing in lines 262-267 to clarification.

- *The results of the fitted zero-inflated Poisson regression model for the various periods are not shown in the manuscript. The results of the Negative Binomial regression model for the various periods are not shown in the manuscript. The criteria values (residual variance, AIC, and BIC) generated by the different models should be presented in the manuscript.*

Reply: The authors are grateful for the observation. In the statistical analysis, the 1,721 municipalities were controlled for their random effects and hierarchically analyzed in terms of state and region to which they belonged. The multilevel model allows joint evaluation of different municipalities and periods, controlling, through random effects, their regional, state and municipal differences, since disease control policies are similar at the regional level, especially in endemic locations. By considering a multilevel model, it was possible to use the information from all municipalities of the 3 surveys (n=1721) in the analysis, improving estimation of the coefficients of the independent variables as well as their respective standard errors and p-values.

We carefully consider the various particularities of each of the three periods of the study. In addition, the period of the study was included in the statistical analyzes as a factor (fixed effect). To support the results of the joint analysis (multilevel model) presented in the paper, the results of a sensitivity analysis were introduced in the Supplementary File S3. Basically, statistical models were adjusted using the data set composed of 41 common municipalities among the three surveys. The model with best fit was the Poisson with zero-inflation and composed of the same explanatory variables as the multilevel model presented in Table 4 of the manuscript. The estimates of the coefficients and Rate Ratio (RR) are similar and, therefore, the results are consistent with those found in the multilevel model presented in the paper.

We also include the goodness-of-fit measures of the adjusted models for the schistosomiasis data, in Table S3.1 of supplementary note S3. The criteria values for Poisson with zero inflated was inserted in Table 4 in the revised manuscript.

We clarify that the purpose of the study is analyze all period of surveys jointly, not each separately. This explanation is inserted in lines 329-341, 344-356 and 535-546.

- *It is critical to use the same number of decimal places for all values in the manuscript. For example, the authors used two decimal places in table 3 but only one in the explanations.*

Reply: We thank the observation and harmonized the decimal places accordingly.

- *Line 286, the value described is not correct [758 (44.0%)]. It should be 788 (45.78%).*

Reply: We thank this remark and revised the value. We apologize for this. Due to wrong typing, the value correct is 758 (sum of municipalities in the 9 states of Northeast). The correct sum is 418 + 114 + 226 = 758, not 788.

The value in table 3 has been changed from 144 to 114. Thus, the absolute value and percentage in the text are correct.

- *The percentage of the subtotal in table 3 for the Northeast region from 1947 to 1952 is incorrect, also the number of municipalities from 1975 to 1979 (114 vs 144).*

Reply: We apologize for this, due to wrong typing, as commented above, the correct value correct is 114, not 144.

- *Line 288, the Northeast and Southeast regions had the highest percentages of municipalities in each survey. This statement should be rewritten as “The Northeast region had the highest percentage of municipalities in each survey following by the southeast……”*

Reply: The sentence was changed accordingly, in lines 315-316.

- *The results are not well explained. The results are not well discussed.*

Reply: The results were further improved in the revised manuscript and supplemental materials. We have also improved the Discussion section, considering the comments from the reviewers. This explanation is inserted in lines 305-309, 329-341, 344-356 and table 4. For Discussion section the explanation is inserted in lines 386-389, 404-411, 416-421, 426-431, 453-456, 525-546 and 559-552,

- *The references are not organized at all.*

Reply: Inserted reference in line 562 and revised all of them, as outlined in the ICMJE sample references.

- *The abstract mentions the prevalence of schistosomiasis was mediated by environmental factors – the analysis method was not a mediation analysis, so I’d suggest the authors revise their phrasing here to avoid confusion*.

Reply: changed to“…associated with…” (lines 22).

**Reviewer3**

- *It’s possible the information on the surveys is lacking, particularly for the second survey, but it’s unclear what the sampling strategies were for selecting municipalities, schools, and children from the population of eligible regions. Was there any random selection of municipalities or schools within them? Were all students at the selected schools examined or was there a sampling strategy applied to students? If it was all non-probabalistic, what methods were used to select/include municipalities/schools/students?*

Reply: PECE occurred in the municipalities where the program had been implemented in that period, by determination of the Ministry of Health, from endemic and non-endemic areas. The criteria for inclusion of schools and students were obtained through census surveys and an active search in school classes. The results of this survey informed the definition of pilot areas for the implementation of integrated measures such as chemotherapy, application of molluscicides and health education. Although we could not find a comprehensive description of the sampling criteria, we inserted this information in the main text (lines 134-138), in Figure 1, and two references were added [13,14] to support the details listed here.

- *I believe the analysis methods used are appropriate, but the explanation is a bit confusing. It is not clear what the response is in the Poisson regression models – the authors state it is prevalence, but Poisson models generally use count as a response and then persons (or person-years) at risk as an offset for this kind of data. This is quite confusing as the authors talk about prevalence in the methods and then talk about rate ratios in results but there does not seem to be a distinction in the models fitted and the response used. For me a prevalence and rate would have a different response variable in the model. I think the outcome variable needs to be made clear in these instances and/or the language (prevalence or rate/rate ratio) needs to be tightened up. Maybe these models could be more explicitly described in an additional supplementary file.*

Reply: The authors are grateful for the observation. The main response variable considered in the statistical analysis is a count variable. For each of the 1,721 municipalities the number of positive results for schistosomiasis among children sampled from the municipality is available as well as the respective prevalence, which is defined as the ratio between the “Number of students with positive stool tests”and the “Total number of students aged 7-14 examined in the respective municipality”. In the statistical model the Poisson and Negative Binomial distributions were used to describe the probabilistic pattern of the count variable for schistosomiasis and the respective number of sampled children was the exposure variable (offset). The link function was the logarithm of the mean (the Poissonand Negative Binomial distributions are part of the exponential family). Therefore, with this modeling it is possible to estimate prevalences as well as the number of positive cases for each municipality, including explanatory (independent) variables to explain the response mean.

Because of the larger amount of municipalities with zero cases of schistosomiasis (45.4% for 1947-1953 period; 54.2% for 1974-1979 period and 73.7% for 2010-2015 period), models with the adjustment for excess of zero were employed in order to verify the robustness and consistency of the analyses. This explanation was insert in lines 11-13, 290-291 and 329-332. In this approach it is possible to adjust a regression model which considers the frequency of zero generated by the Poisson (and Negative Binomial) as well as the frequency of zero that is generated by another distribution related to the excess of zero. Although there are many distributions that can be used with this purpose, the logistic distribution is very common. In the adjustment of zero inflated models, two probability distributions (Poisson (or Negative Binomial) and Logistic) are combined to generate estimates of the number of cases and prevalences for each sample unit (municipality) considering the respective values of the explanatory variables included in the models.

For each quantitative independent variable, the RR presents the ratio between the average prevalences of positive cases of the Schistosomiasis considering a situation of an increase of 1 unit of measurement in the respective independent variable.
Thus, it is an estimate of the change of the average prevalence as the independent variable increases by an additional unit, being the calculation performed considering that all the other independent variables included in the model are fixed. The maximum likelihood statistical procedure was used to estimate the parameters of the models.

We included the discussion above in the Supplementary File S3 and also a comment in lines 258-261 and 365-366 of the revised manuscript. Table 4 was modified to include results on the zero-inflation part of the model.

- *There is a large amount of ‘missing’ data in the longitudinal models resulting from the changing survey sampling methods and I would like to see some consideration and discussion of this in the manuscript. Of the over 1,700 municipalities included in the longitudinal analysis, only around 400 municipalities had repeated survey data and so the sampling methodology is not necessarily consistent with conclusions that prevalence is decreasing. The authors could consider including a sensitivity analysis that includes only the municipalities with 2 or 3 surveys to see if those longitudinal patterns are similar to patterns for the full sample*.

Reply: We thank the reviewerfor this valuable suggestion. We agree that the addition of this information would be interesting. We reiterate that in this case, the total number of municipalities common to the three surveys was small (n = 41), which would not allow a robust statistical analysis, such as that carried out with the entire data set in this article. However, we considered it necessary to include this consideration in the section on the results and discussion of the work, therefore, it was included in both sections.“Additionally, for the 41 municipalities common to the three surveys, the percentage decrease in prevalence between the 1947-1953 survey and the 2010-2015 survey ranged from 0.1 percentage point (p.p) to 77.4 p.p., with only three municipalities presenting small positive percentage difference between 0.4 and 0.1 p.p.”(lines 305-309). In the discussion section (lines 387-389): “The descriptive analysis among the municipalities common to the three surveys indicated a decrease in the prevalence percentages for most of the analyzed municipalities (92.7%)”.

As suggested, a sensitivity analysis were introduced in the Supplementary File S3.Basically, statistical models were adjusted using the data set composed of 41 common municipalities among the three surveys. The model with best fit was the Poisson with zero-inflation and composed of the same explanatory variables as the multilevel model presented in Table 4 of the manuscript. The estimates of the coefficients and Rate Ratio (RR) are similar and, therefore, the results are consistent with those found in the multilevel model presented in the paper. As an advantage. The multilevel model allows evaluating different municipalities and periods, controlling, through random effects, their regional, state and municipal differences, since disease control policies are similar at the regional level, especially in endemic locations. The multilevel model provides better inference from grouped data.

Also, it was included the results of a statistical analysis comparing the distribution of the municipalities of the 3 surveys according to the endemicity classification used in the sampling procedure of the third survey (2010-2015). The results indicated that, although there are differences in the form of data collection regarding the selection of municipalities, the 3 samples are comparable in terms of the endemicity degree criterion used in the 2010-2015 survey. This explanation was inserted in lines 344-356.

- *The discussion of the findings is detailed and thoroughly linked to other relevant literature. The authors have discussed how the findings can be used to improve public health outcomes for children. I'd like to see a sensitivity analysis (see previous comment) to help support the conclusions and the limitation of missing data should also be discussed*.

Reply: The results of a sensitivity were included in the Supplementary material. Basically, statistical models were adjusted using the data set composed of 41 common municipalities among the three surveys.

The model with best fit was the Poisson with zero-inflation and composed of the same explanatory variables as the multilevel model presented in Table 4 of the manuscript.The estimates of the coefficients and Rate Ratio (RR) are similar and, therefore, the results are consistent with those found in the multilevel model presented in the paper. It was also included the results from a statistical analysis comparing the distribution of the municipalities of the 3 surveys according to the endemicity classification used in the sampling procedure of the third survey (2010-2015). The results indicated that, although there are differences in the form of data collection regarding the selection of municipalities, the 3 samples are comparable in terms of the endemicity degree criterion used in the 2010-2015 survey.

- *The abstract mentions the prevalence of schistosomiasis was mediated by environmental factors – the analysis method was not a mediation analysis, so I’d suggest the authors revise their phrasing here to avoid confusion*.

Reply: term replaced by “associated with” (line 22).

- *In Table 1, the description of the literacy rate doesn’t include how literacy was defined in the numerator and currently only states “Number of people aged 15 years old or Older”.*

Reply: Changed, including the word “literate” in the numerator.

- *Line 199 should say explanatory variable not exploratory.*

Reply: Revised, thanks. (line 197)

- *Line 244 should say generalized linear mixed models not generalized mixed linear models to align with the acronym GLMM*.

Reply: Revised, thanks. (lines 246-247)

- *Table 2 and associated text - the term ‘range’ is usually used for the difference between maximum and minimal values.*

Reply: Revised, thanks.

- *Line 350 "the study proves" is very strong language and should perhaps be modified to something along the lines of 'the study has found significant associations between environmental factors and schistosomiasis'*

Reply: Modified according to suggestion (line 386).

**Reviewer4**

- *The surveys from which the data are sourced are clearly explained. However, a major concern is that the three surveys all seem quite different. This raises the question of how appropriate it is to use these three separate datasets in a longitudinal approach (different measures are used in each survey and different sampling techniques).*

Reply: Dear reviewer, we appreciate the valuable comments and agree that some different approaches were applied in the surveys. We acknowledge that there are limitations in our study. However, we emphasize that all the peculiarities were not disregarded. we support that analysing 3 large surveys, covering such a large time span with large samples is an opportunity for exploring the data and extracting possible findings of the association between environmental conditions and disease prevalence. For the sample of the second survey, it consisted of a sample from endemic and non-endemic areas for the disease, with criteria for defining schoolchildren, schools, and municipalities such as the 1947-1952 survey. We believe that, even the sample being not probabilistic, a robust criteriafor surveying and collecting data was applied, not compromising the estimates of disease prevalence.Regarding the parasitological tests, during the 1947-1952 IHE, Brazil had a high prevalence of schistosomiasis and a high intensity of infection, implying that the application of less sensitive diagnostic methods, such as the technique of spontaneous sedimentation in leads to a low number of false-negative. The results obtained in the two last surveys, could identify a greater number of true positive, with a low detection of false-negatives, due to superior sensitivity of the methods.

These points are due justified and the main text has been modified to make the points as clear as possible:modification in the writing of the introduction about the surveys; in figure 1; item 2 in the subtopic "studies period and data source" - in methods (lines 134-138); discussion about the different diagnostic techniques (lines 525-534) and addition statistical analysis, as below.

The applied statistical analysis supports the structure of the data and allowed revealing important results not yet studied in the country, considering the representativeness of a national sample and with historical temporality.The option of using multilevel model is based on the fact that it allowed to use the information from all municipalities of the 3 surveys (n=1721) in the analysis and improving the estimation of the parameters of the model, respective standard deviations and p-values.It is well known that multilevel models(withrandom effects) provide better inference from grouped data (in the case of the presented study, students are grouped in municipalities which are grouped in states which are grouped in regions) since the coefficient and variance error for each explanatory variable are better estimated, avoiding the problem of underestimation of coefficients and overstatement of their significance that occur when clustering effect is not taken into account. This explanation was inserted in the manuscript in “discussion”, lines 535-546.

Also, the results of a sensitivity analysis were introduced in the Supplementary File S3. Basically, statistical models were adjusted using the data set composed of 41 common municipalities among the three surveys. The model with best fit was the Poisson with zero-inflation and composed of the same explanatory variables as the multilevel model presented in Table 4 of the manuscript.The estimates of the coefficients and Rate Ratio (RR) are similar and, therefore, the results are consistent with those found in the multilevel model presented in the paper. It was also included the results from a statistical analysis comparing the distribution of the municipalities of the 3 surveys according to the endemicity classification used in the sampling procedure of the third survey (2010-2015). The results indicated that, although there are differences in the form of data collection regarding the selection of municipalities, the 3 samples are comparable in terms of the endemicity degree criterion used in the 2010-2015 survey. The last paragraph was included in the manuscript in “Results”, lines 344-356.

- *Line 10: grammatical issue*: changed ‘was examined’ to ‘were examined’.
- *Line 12: Unclear on the meaning of ‘were adjusted’. I expect a phrase like ‘were estimated’ here*.

Reply: All the above have been fixed according to suggestions (lines 9-11).

- *Line 22: Unclear on the meaning of ‘other ways of contact with watercourses’. Thisphraseis a littleclunky.*

Reply: changed to “other ways of contact with unsafe water bodies” (line 24).

- *Line 38: ‘momentum historical’ doesn’t make sense*.

Reply: excluded the term momentum. Changed to “Thus, national surveys on the national prevalence of the disease covering a large time span can provide valuable information about its epidemiological pattern over the years".(lines 40-41)

- *Line 46: The part of the sentence that comes after the comma doesn’t seem to fit with the first part of the sentence. You appear to say that prevalence of schistosomiasis decreased, but that this suggests that better approaches are needed for controlling the disease.*

Reply: changed to “The results showed that the decrease in schistosomiasis from the 1950s to the 2010s was statistically significant and suggests that improvements in water supply and sanitation require structured and systemic approaches for controlling the transmission of schistosomiasis.”(lines 47-50)

- *Line 71: Wording issue: ‘control measures conditions’. Unclear what you are trying to say in this phrase*.

Reply: changed to “In Brazil, the epidemiology of *Schistosomamansoni* infection shows that social and, environmental conditions, drug treatment and access to health service contribute to a reduction in the prevalence rate [5]”.(lines 76-78)

- *Lines 84-100: In this section, you examine how the prevalence rate has changed over time. However, I am concerned as to whether this comparison is valid as it appears that each survey targeted a different Brazilian sub-population. Also, for the surveys used, it appears that different sub-populations were the target of each survey. Thus, it is difficult to understand what population the authors are attempting to understand with the sample they have.*

Reply: this introductory text intends ofunderline characteristics of the surveys, and it was shortened for clarity (lines 91-98). The population included in the three surveys has common features, i.e, students between 7 and 14 years of age enrolled in the education teaching network in Brazilian schools. The small differences observed between the prevalence values described in the removed text and the results were due to the inclusion and exclusion criteria of municipalities and not to sub-samples of municipalities.

- *Line 110: The wording ‘a longitudinal study on three panels’ sounds a little odd. Perhaps it would be clearer to say something like: a longitudinal study covering three waves of data.*

Reply: changed to “The epidemiological design of the research consists of a prospective study, covering three panels with observational ecological data” (lines 110-111).

- Line 126: The word ‘besides’ is unclear in this context.

Reply: The sentence and word was removed for clarification, because these cities would be included in the sample (lines 125-129).

- *Line 144: By stating that there were 197,564 examinations of schoolchildren, it may suggest to the reader that the unit of observation is at the child level (whereas it is at the municipality level). Perhaps it would be more helpful to focus on whether a representative sample of children were examined in each municipality as well as comment on the numbers involved.*

Reply:modified writing to emphasize the unit of analysis used, that is, the municipality. “As a result, 521 municipalities representing all Brazilian states were conducted, the percentage of tests performed ranged from 60% to 100% and in nine states it was higher than planned. In total, 197,564 schoolchildren were examined” (lines 147-150).

- *Line 146-160: In this section, the authors describe differences in the techniques used in the different surveys: different methods to measure prevalence and even different age ranges*.

Reply: We have adjusted the text to make it clearer regarding differences in age groups. We emphasized that the inclusion of age groups from 15 to 17 did not result in a statistically significant difference in the sample, according to the description of the methodology and detailed results of INPEG (KATZ, 2018), and for this reason they were maintained in this survey. We believe it is pertinent to remove this information to contribute to the clarity of the text and better understanding of the methods, but in supplementary material S1 also contain this explanation.

Katz N. Inquérito Nacional de Prevalência da Esquistossomose mansoni e Geo-helmintoses. Belo Horizonte: CPqRR; 2018. 31-37p. (Série Esquisstossomose). Available in: <http://tabnet.datasus.gov.br/cgi/sinan/inpeg/RelatorioINPEG.pdf>

- *Line 161: Figure 1 is very clear for the most part. There are a coupe of places where it looks like the wording has not been translated  into English: ‘Alunos Examinados’?*

Reply: the figure was changed and words has been translated. Thanks.

- *Line 169, Table 1: The description of the prevalence of schistosomiasis measure raises some concern. The numerator used to calculate the prevalence is the number of students with a positive stool sample. However, earlier the authors suggested that different approaches to the stool testing have been used in different surveys. E.g., the Kato-Katz method was not used in all surveys. The denominator used to calculate the prevalence also raises some concerns as it is unclear what population is represented by the sample of 'all examined students'. This comment echoes concerns in relation to earlier parts of the article on how appropriate it is to compare and combine all waves of data.*

Reply: the numerator, for both diagnostic tests used in the surveys – Hoffman and Kato-Katz, consists of positive tests for Schistosoma mansoni eggs. The denominator was the amount of students examined in each survey. To clarify this, a modification was made to the description of the prevalence reported in Table 1 – denominator “total number of students aged 7-14 years examined”.

This explanation was inserted and moved from “methods” for “discussion”. (Line 525-534): “Regarding the parasitological tests used in the surveys, although two different methods were used, the comparability between them is possible. Firstly, during the 1947-1952 IHE, Brazil had a high prevalence of schistosomiasis and a high intensity of infection, implying that the application of less sensitive diagnostic methods, such as the technique of spontaneous sedimentation in water (Hoffman, Pons et al. Janer, or HPJ technique) [70], leads to a low number of false-negative [71]. Secondly, the results obtained in the two last surveys (PECE and INPEG), which used the Kato-Katz method, could identify a greater number of true positive, with a low detection of false-negatives, due to superior sensitivity of the method. It is well known that the Kato-Katz method is currently the gold standard method recommended by the WHO [3,72]”. We emphasize that, in 1950, have used less sensitive test, as HPJ, could detect low number of false-negative, since Brazil had a high prevalence and intensity infection in that period.

- *Line 169, Table 1: I believe the literacy rate description is missing some words. You need to specify that the numerator includes only people age 15 or older 'who are literate'?*

Reply: changed, including the word “literate” in the numerator.

- *Line 204-205: Unclear what is meant by the phrase ‘seeking to adjust the uniformity and consistency over time’*.

Reply: we agree that it was not clear and that it was excessive a placement since before the previous sentence it is considered that the variables were defined based on these two criteria: according to availability and that they could be compared between the three surveys, simultaneously. We chose to withdraw (line 200-202).

- *Line 213 – 229: It is a little concerning that so many of the key explanatory variables are constructed using projections. I appreciate the authors transparency on this. Perhaps if they presented a table or outlined in the writing how many observations are affected, that would help to shed light on the likely implications of this*.

Reply: Added information to the main text that refers to the descriptive table (item 2 Supplementary Material S2 and inserted in lines 228-230 in the manuscript) about the techniques used and the number of observations. The authors clarify that the decennial measures of the demographic censuses of the years 1980 or 2010 could be used for the years of each surveyfor the explanatory variables, without using projection techniques. However, we chose to perform the average approximation estimate with the response variable of the study (1977 and 2013). The techniques are widely used, including for studies with more recent data as a way of more accurately estimating the variable for the year of the study.

- *Line 231: Unclear what is meant by the statement ‘the national surveys are not continuous’*.

Reply: means that, since 1950, the three surveys used in this research were the only ones carried out in Brazil, with national representation. Changed to “As the national surveys were carried out in different periods and using different strategies,”(line 232-233).

- *Lines 243-257: The discussion of the GLMMs lacked clarity. There is a lot of discussion in this section about modelling of ‘data’, without any specific discussion of the relevant variables. E.g., in the discussion of random effects, it is unclear what variable this specifically refers to. It would be useful to clarify.*

Reply: we thank the reviewer for the suggestion. In order to clarify, we modified the text in lines 245-253– to the following and also made it clear in Table 3.

“In order to consider this hierarchical characteristic of the data, we applied Generalized Linear Mixed Models (GLMMs) with random effects related to three levels: regions (Level 1, with 5 categories South, Southeast, North, Northeast and Midwest); states (Level 2, representing the 26 federative units plus the Federal District); and municipalities (Level 3, related to the 1,398 different municipalities included in the study with observation in at least one of the three surveys). These three hierarchical levels of data were incorporated into the random intercepts of the GLMMs to allow the joint modeling of data from the different municipalities of each sampling period”.

- *Lines 259-261: In explaining the reason for selecting the final model, the authors mention using the AIC, BIC and residual variance criteria. It is unclear how comparable these criteria are for differenft models (Poisson vs Negative Binomial) and with different degrees of freedom. Also, do all 3 criteria consistently point to the same model? Could one criterion point to one model being most appropriate while another criterion points to another model? It would be helpful to pre-empt these questions in the discussion of the process used for model selection*.

Reply: In orther to clarify the selection criteria, we modified sentence in lines 267-269 to “The final regression model (Poisson or Negative Binomial distribution) was chosen according to the lowest residual variance and then consideration of zero inflation was defined with basis on the lowest values for the Akaike Information Criterion *(*AIC) and the Bayesian Information Criteria (BIC).” The results between the final adjusted models (Poisson and Negative Binomial models, with and without zero inflation) can be verified in Supplementary File S3.

- *Line 301-302: It would be helpful to offer a little more clarity on how many models were considered. That could be discussed here, or earlier in the article.*

Reply: we thank the comment and included additional information where appropriate. In Methodology, we inserted “Logistic distribution was used to adjust for the excess of zero” (lines 260-161). In Results: “The goodness-of-fit analysis of the models was performed using the following statistics: lower values of residual variance, AIC, and BIC. The results between the final adjusted models can be verified in Supplementary File S3. In the comparison of Poisson and Negative Binomial models, the residual variance was smaller for Poisson models being the zero-inflated the model with the lowest AIC and BIC values. Therefore, the zero-inflated Poisson model presented the best performance.” This explanation was inserted in lines 332-338.

- *Lines 301-305: I think it would help the reader to understand the coefficients and RRs presented in the model if the equation of the model you are estimating was explicitly stated*; *To help readers less familiar with the research on disease prevalence rates, it would be helpful to note somewhere that this is the standard approach used to examine disease prevalence. Similarly, the authors do not explain the specific implications of the ‘zero-inflated’ Poisson regression*.

*In relation to the statistical analysis, the article lacks a clear foundation and motivation for the techniques employed. The authors note that they use a Poisson regression. To help readers less familiar with the research on disease prevalence rates, it would be helpful to note somewhere that this is the standard approach used to examine disease prevalence. Similarly, the authors do not explain the specific implications of the ‘zero-inflated’ Poisson regression. For example, no equations are presented to help the reader understand what equation is being estimated.*

Reply: The authors are grateful for the observation. The main response variable considered in the statistical analysis is a count variable. For each of the 1,721 municipalities the number of positive results for schistosomiasis among children sampled from the municipality is available as well as the respective prevalence, which is defined as the ratio between the “Number of students with positive stool tests”and the “Total number of students aged 7-14 examined in the respective municipality”.

In the statistical model the Poisson and Negative Binomial distributions were used to describe the probabilistic pattern of the count variable for Schistosomiasisand the respective number of sampled children was the exposure variable (offset). The link function was the logarithm of the mean (the Poissonand Negative Binomial distributions are part of the exponential family). Therefore, with this modeling it is possible to estimate prevalences as well as the number of positive cases for each municipality, including explanatory (independent) variables to explain the response mean.

Because of the larger amount of municipalities with zero cases of schistosomiasis (45.4% for 1947-1953 period; 54.2% for 1974-1979 period and 73.7% for 2010-2015 period), models with the adjustment for excess of zeros were employed in order to verify the robustness and consistency of the analyses. This explanation was insert in lines 11-13, 290-291 and 329-332. In this approach it is possible to adjust a regression model which considers the frequency of zero generated by the Poisson (and Negative Binomial) as well as the frequency of zero that is generated by another distribution related to the excess of zero. Although there are many distributions that can be used with this purpose, the logistic distribution is very common. In the adjustment of zero inflated models, two probability distributions (Poisson (or Negative Binomial) and Logistic) are combined to generate estimates of the number of cases and prevalences for each sample unit (municipality) considering the respective values of the explanatory variables included in the models.

For each quantitative independent variable, the RR presents the ratio between the average prevalences of positive cases of the Schistosomiasis considering a situation of an increase of 1 unit of measurement in the respective independent variable.
Thus, it is an estimate of the change of the average prevalence as the independent variable increases by an additional unit, being the calculation performed considering that all the other independent variables included in the model are fixed. The maximum likelihood statistical procedure was used to estimate the parameters of the models.

We included the discussion above in the Supplementary File S3 and also a comment in line 254-261, 332-338, 344-353 of the revised manuscript. Table 4 was modified to include results on the zero-inflation part of the model.

- *Line 274, Table 2: In the notes about the GDP measure, I expected it to state the base year for the GDP measure (as Table 1 stated that GDP is measures in constant R$).*

Reply: GDP at constant prices for the year 2000 (thousand) was used; information entered in Tables 1 and 2 (lines 164, 297).

- *Line 308. Table 4: I wonder should the estimate for ln(population) read as 0.138 rather than 0.148? In the writeup you state than the associated effect is a 13.8% decrease? If the explanatory variable is in logs, I believe this effect can be interpreted as an elasticity?*

Reply: The interpretation of 13.8% decrease is related to the RR associated to ln(population). The point estimated for its coefficient is in fact -0.148 which leads to a RR=exp(-0.148)=0.862 (line 419).

- *Line 323: Typo – change 32.5% to 35.2%.*

Reply: changed to 35.2%. Thanks (line 375).

- *Lines 355-362: I found the writing in this section to be unclear*.

Reply: Changed to make it clearer.“For instance, a significant association was found in households that did not have any type of sewage disposal when compared to families using a safe sewage network (OR 1.8; CI 1.3 – 2.4) [26]. Additionally, national and international studies showed that the improvement of the sanitary installation for domestic wastewater collection was significantly associated with a decreased probability of infection [27,28] (lines 404-409).

- *Line 384: It is unclear how the 27.4% has been calculated.*

Reply: according to the example cited, considering the difference of 80% between the lowest and highest percentage of coverage, if the variable collection network has an increase of 80 units, we would have exp(-0.004*80) = 0.726; that is, we estimate a reduction of approximately 27.4%. A short description has been added to the text (line 419). This is related to the calculation of RRs for Poisson regression coefficients.

- *Line 415-418: It is unclear what point is being made in the sentence beginning ‘An intervention study…’*.

Reply: the impact of using safe sources is only noticeable when piped water is continuously available and in sufficient quantity for the user, otherwise there is no complete adherence to safe use. The textual construction has been changed for better clarification. “An intervention study showed that the positive impact of piped water occurred only when the amount of water available was higher than 1,000 liters per person per year, ie, the use of unsafe water can continue if only a small amount of water is provided or if there are interruptions due to precarious distribution systems [45]” (lines 453-456).

- *Line 489: The authors state that the findings from this research show ‘that the reduction in the prevalence of schistosomiasis in Brazil over seven decades can be explained by ….’ But later in line 505, the authors state the results cannot be generalized outside of the specific sample. Later on line 519, the authors also state that the prevalence decreased significantly over the decades in Brazilian municipalities. There is a lack of clarity on whether these results should be used to make inference about the entire Brazilian population or if the results cannot be generalised outside of the sample*.

*The population is not very clearly described. In parts of the articles, the authors suggest that the results allow for inferences to be made about the impact of environmental factors on schistosomiasis in ‘Brazil’. However, in the Limitations section, the authors state that the results are only informative about this sample – this contradicts the suggestion that the results are generalisable to ‘Brazil’.*

Reply: although the sample was made up of different municipalities and in different periods, in all surveys representative municipalities from endemic and non-endemic areas were included, with strong national representativeness. For this reason, the authors considered these particularities in the statistical analysis and discussion of the results, and we are confident to state the external validity of the study. The text has been improved (line 564-567).

- *Line 524 – 526: The authors make recommendations about offering education on sanitary sewage and health. It is unclear how this recommendation emerges from this study.*

Reply: changed to include only the recommendation to offer sanitary sewage, especially the collection network, a condition evaluated in this study (line 586-590). “In addition, safe sanitation sewage should be widely provided to the population at the household level and other spheres of life, such as workplaces, health centers and school environments. Other national prevalence surveys and research should be conducted more continuously to monitor the disease prevalence and its determinants over time”.

We hope to have addressed all reviewers' concerns appropriately and respectfully, and we reiterate our thanks to the reviewers for their unvaluable assessment. We remain available for any further clarification.

Belo Horizonte, Brazil, January 12, 2023

Mariana Cristina Silva Santos

Guilherme Lopes de Oliveira

Sueli Aparecida Mingoti

Léo Heller
