# Supplementary material for: Effect of environmental factors in reducing the prevalence of schistosomiasis in schoolchildren: An analysis of three extensive national prevalence surveys in Brazil (1950–2018)
Source: PLoS Negl Trop Dis. 2023 Jul 17;17(7):e0010804. doi: 10.1371/journal.pntd.0010804 (PMC10374055; doi:10.1371/journal.pntd.0010804)
Supplement: Attachment — Submitted filename: Letter to the Review Editor PNTD 07 maio.docx [file pntd.0010804.s007.docx]

**LETTER TO REVIEWERS**

Dear Editor,

We thank again the reviewers for the valuable observations and suggestions for improving our manuscript, which have greatly contributed to the quality of this new version. We tried to accommodate most of the suggestions, certain of their relevance and appropriateness. The changes and additions made are highlighted in the text file sent and in this letter. When the comments were not clearly described in our view, we addressed them based on our understanding. Please find below our point-by-point comments.

**Reviewer 1**

Thank you for all the valuable suggestions which have improved the paper’s adequacy and quality.

- ***Methods (minor comments):***

1) *The use of P-value: Pearson's Chi-squared statistical test comparing the period with 2013 in S3 - supplementary material is fundamental for validation of this research. Please, clarify this state in the S3 - supplementary material: “As shown in Table S3.3, the p-values of the tests indicated that the distributions were similar to the third survey (p-value=0.598 for 1950; p-value=0.183; for 1977; p-value=0.145, when considering common municipalities in all three surveys). Considering these results, it can be concluded that, although there are differences in the form of data collection regarding the selection of municipalities, the three samples are comparable in terms of the endemicity degree criterion used in the 2011-2015 survey.”*

**Reply**: We thank the reviewer for raising this point. To clarify the performed analysis, we rewrite the referred paragraph and Table S3.4 as follows:

Although the classification (or definition) of degree of endemicity may have changed from 1947-1953 to 2010-2015, the distribution of the endemic levels from 2010-2015 survey was compared with the respective distributions from 1947-1953 and 1975-1979, using the following procedure. First, the municipalities that were sampled in both 1947-1953 and 2010-2015 periods (same municipalities) were identified and associated with the respective endemic classification (Non-endemic, Low-prevalence, High-prevalence) according to the information available in the 2010-2015 period. Then, the number and proportion (percentage) of municipalities at each endemic level were calculated. Finally, the distributions of proportions observed in 1947-1953 and 2010-2015 were compared using Pearson's Chi-square statistical test. The same procedure was carried out for the 1975-1979 data.

As shown in Table S3.4, the p-values of the tests indicated that the distributions were similar (p-value=0.183 for comparison between 1975-1979 and 2010-2015; and p-value=0.598 for comparison between 1947-1953 and 2010-2015). Considering these results, it can be concluded that, although there are differences in the sampling procedures, the three samples are comparable in terms of the proportion of municipalities within each endemic level with basis on the endemicities reported in 2010-2015 survey”.

**Table S3.4: Distribution of endemic levels between surveys**

| **Period** | **Endemicity Level** | | | **P-value** |
| --- | --- | --- | --- | --- |
|  | **Non-endemic n (%)** | **Low-prevalence n (%)** | **High-prevalence n (%)** |  |
| 2010-2015 | 372 (71.4%) | 85 (16.3%) | 64 (12.3%) | - |
| 1975-1979 | 34 (56,6%) | 13 (22,8%) | 10 (17,6%) | 0.183 |
| 1947-1953 | 98 (67.1%) | 28 (19.2%) | 20 (13.7%) | 0.598 |

P-value: Pearson's Chi-squared statistical test comparing the period with 2013. n: Total number of sampled municipalities.

- ***Editorial and Data Presentation Modifications***

2) *Abstract: please review the recommendations and, if possible, some of the suggestions.*

**Reply**: In line 19 we added the suggestions, i.e., “association with the piped water supply”.

3) *We noted that it is necessary to carefully review some editorial issues (like the lines 18, 62, 88) before submitting the final version of the article.*

Reply: Thanks for this contribution. Errors were corrected and suggestions were incorporated (lines 19, 60-61, 82-93).

**Reviewer 2**

***Summary of strengths, weaknesses, and overall contribution:***

*Thank you for sending me back the article.*

*From my perspective, I raised some comments/suggestions, which are not all addressed. The different observations are listed below.*

**Reply:** Thank for all valuable suggestions which have improved the paper adequacy and quality. We hope all of your concerns are clarified now.

1. *A map of Brazil showing the administrative divisions (regions) and/or a description of how the administrative regions differ from states (level 2) and municipalities (if possible) would be helpful in explaining some of the differences in prevalence (as you mentioned in the methodology that the municipality as the unit of analysis).*

**Reply**: We appreciate the suggestion and inserted a description on how the administrative regions differ from states and how they were considered in our analysis (lines 205-215). “According to the official territorial division proposed by the IBGE, the Brazilian political-administrative organization is divided into five macro-regions, which include 26 federal units (states) plus the Federal District and 5,570 municipalities. In order to consider this hierarchical characteristic of the data, we applied Generalized Linear Mixed Models (GLMMs) with random effects related to three levels: regions (Level 1, with 5 categories South, Southeast, North, Northeast and Midwest); states (Level 2, representing the 26 federative units plus the Federal District); and municipalities (Level 3, related to the 1,398 different municipalities included in the study with observation in at least one of the three surveys). These three hierarchical levels of data were incorporated into the random intercepts of the GLMMs to allow the joint modeling of data from the three periods”.

2. *A backward selection procedure was used to identify the significant fixed effects, considering a 25% significance level for the removal of an explanatory variable. Why the authors considered 25%?*

**Reply**: Setting a higher significance level in the univariate analysis is strongly recommended in backward selection in order to allow more variables to be included in the final model. The usual cut-off significance level of only 0.05 in this stage can sometimes exclude important variables in the process of variable selection. After removing variables using this higher cut-off level, a new process of selection is applied in a more detailed analysis that may possibly indicate the removal of new variables. In our study, after the first stage, all remaining variables were already significant at 5%. We rewrote the sentence in lines 225-233 for better clarification.

“A backward selection procedure was used to identify the significant fixed effects, considering a 25% significance level for the removal of an explanatory variable. Thus, at each step of the analysis, the explanatory variable with the highest p-value, among those with a p-value>0.25, was removed from the model. After such a procedure, no further variable selection was necessary, as all variables retained were significant at the 5% significance level. The final regression model (Poisson or Negative Binomial distribution with or without zero inflation) was chosen according to the following criteria: (a) lower residual variance; (b) lower values of Akaike (AIC) and Bayesian (BIC) Information Criteria”.

3. *The results of the Negative Binomial regression model for the various periods are not shown in the manuscript. 4- The criteria values (residual variance, AIC, and BIC) generated by the different models should be presented in the manuscript.*

**Reply**: The authors are greatful for the observation. We clarify that the purpose of the study was to analyze all periods of surveys jointly, not each separately. Multilevel Poisson and Negative Binomial models with and without excess of zeros were adjusted to the data. The criteria values for Poisson with zero-inflated, chosen as the best fitted model, were inserted in Table 4 of the revised manuscript. Criteria values (residual variance, AIC and BIC) for all Poisson and Negative Binomial fitted models, with and without excess of zero, were included in Table S3.1 of the Supplementary File S3 (Supplementary Statistical Analysis). We found it convenient to keep in the main manuscript only the details and results related to the best model, as the discussions are based on it. Nevertheless, explanation about steps and criteria for the best model selection were edited in Lines 231-233 and 296-302 for clarification.

4. *Line 312, the value described is not correct [758 (44.0%)]. It should be 788 (45.78%).*

**Reply**: We thank this remark and revised the value. We apologize for this. There was a typing mistake in Table 3 of the manuscript corresponding to the sum of the values of the PECE (1975–1979) column. The correct value is 114 not 144. Therefore, the sum of municipalities in the 9 states in the Northeast region is: 418 + 114 + 226 = 758, not 788, and the correct percentage is 44% ( line 277).

5. *The percentage of the subtotal in table 3 for the Northeast region from 1947 to 1952 is incorrect, also the number of municipalities from 1975 to 1979 (114 vs 144).*

**Reply**: We apologize for this. Due to typing mistake, as explained in the previous question (item 4), for the sum of the PECE (1975-1979) column in Table 3 is 114, not 144. Please, see response to comment above (item 4).

***Conclusions and Results:***

6. *The analysis presented not match with the analysis plan.
   Tables are not clear. The limitations of analysis are not clearly described.*

Reply: Based on the considerations and suggestions for improvement proposed by the reviewers, more detailed explanations were included in the methods and results sections. Thus, to support the performed analysis, we included a sensitivity analysis by fitting the model to the 41 municipalities common to the three surveys (Supplementary File S3); comparison between the distribution of endemicity levels of the municipalities sampled in the surveys (Supplementary File S3); model predictive analysis (Supplementary File S3); a justification regarding the sensitivities of the parasitological diagnostic methods (lines 476-485); a description of the levels used in the random effects of the models (lines 208-215); and the results for the logistic adjustment for the analysis of excesses of zero in the Poisson model in Table 4. The discussion about limitations of the analysis was modified (lines 486-500). We hope that the matching of planned and performed analysis is clearer in the revised manuscript. Tables and limitations were edited following the reviewer suggestions in order to make them clear.

**Reviewer 4**

Thank you for highlighting the strengths and potential of our work. All your valuable suggestions have improved the paper adequacy and quality.

***Methods:***

*My remaining concerns:*

1. *A) I think the key aspects of the Data Analysis are lost, at times, with convoluted language. I, personally, would find it helpful to see an equation/equations to better explain the methodology (one equation showing the main estimation framework - no need for equations for the sensitivity analysis, model selection process). However, I understand that may not be everyone's preference.*

**Reply:** The authors are grateful for the observation. In order to better describe the methodology, we included details in Supplementary File S3 (Supplementary Statistical Analysis) and also a comment in line 220 and 334-337 of the revised manuscript. Table 4 was also modified to include results on the zero-inflation part of the model. To avoid cumbersome notation involving equations of the generalized linear mixed models (GLMM), with and without zero-inflation, we prefer do not include them, however additional references for those interested in the methods were included in the manuscript and in the Supplementary File S3:

B.M. Bolker, M.E. Brooks, C.J. Clark, S.W. Geange, J.R. Poulsen, M.H.H. Stevens, J.S.S. White Generalized linear mixed models: a practical guide for ecology and evolution Trends Ecol. Evol., 24 (2009), pp. 127-135

B.M.Bolker. Linear and Generalized Linear Mixed Models. In G.A.Fox,S.Negrete-Yankelevich,and V.J.Sosa,editors, Ecological Statistics. OxfordUniversityPress,Oxford,UK, 2015. [p378,379]

1. *B) Similarly, I think that many of the key points about the data are lost due to overwhelming detail on the data selection process etc. To begin with, I think it is unnecessary to provide a rough summary of the data in the introduction before the more detailed discussion in the Methods section which follows. Perhaps refer to it much more briefly in the introduction and explain that the remore detailed discussion will follow. I also think it would be helpful to reduce the discussion of the data selection process somewhat in the main text and keep that finer detail for the supplementary material. I think it would be helpful to move a lot of the more convoluted explanations relating to the choice of model to the supplementary material. This would allow readers to focus on your final chosen model and how to interpret it.*

**Reply**: We appreciate the suggestion and the sentences have been rewritten in the Introduction for clarification (Lines 85-90). We also considered modifications to reduce the discussion about the data selection process in lines 181-194, and removed explanations relating to the choice of model (see line 221). In Results, modifications were carried out in lines 296-302, sensitivity analysis was mentioned in lines 234-237 and 498-500, we rewrite the results of model (line 335-347) for leaving the broader Discussion only in the Supplementary Files S2 and S3.

2. *Line 18: 'inverse association with the water supply' is unclear. You need to indicate whether you mean piped water supply etc to help the author understand if this 'inverse association' represents a favourable or unfavourable association.*

**Reply**: Changed to “inverse association with the piped water supply”. We clarify that the water-related variable used in this study was domestic piped water supply, but no other additional information on disruption, infrastructure conservation or service acceptability was measured. Therefore, we paid close attention to this inverse association in the discussion (lines 383-385) and in the Abstract (line 19).

3. *Line 82: Meaning of 'exclusive' is unclear here. Perhaps you are trying to get across that it can not be treated as a silo.*

**Reply**: Changed to “. Approaches to exploring and understanding the role of environmental, biological and medical interventions, as well as historical, socioeconomic, and cultural determinants, are key of assessing this complex disease.” (lines 78-81). Thanks.

4. *Lines 84-90: You do not indicate immediately here that this survey covered prevalence of schistosomiasis. On line 90 you then say 'the disease' but it not clear what disease you are referring to.*

**Reply**: Changed to “conducting surveys on the prevalence of schistosomiasis” (line 82-83) and “Given the epidemiological and social impact of the schistosomiasis..” (line 85). Thanks.

5. *Line 95-106: I feel this section raises lots of questions for the reader because it skims over the data. I wonder could this part be removed/reduced and save the data discussion for the more detailed section that comes next.*

**Reply**: We agree and repharesed it in lines 82-96. Thanks.

6. *Line 110: I think the word 'periods' or 'waves' would be more appropriate than 'panels'. A panel implies a longitudinal dataset that already consists of multiple periods. If this is what you are trying to communicate, it is fine to leave the writing as it is. My understanding, however, is that each of the three datasets are treated as one midpoint in time.*

**Reply**: Changed to “covering three periods with observational ecological data” and the title of the article (lines 99-100). Thanks,

7. *Line 112: Saying that the 'explanatory variables' represent population groups does not make sense to me. This would only make sense if you were creating subgroups based on the explanatory variables. But I don't think that is what you are trying to communicate.*

**Reply**: The authors agree with this observation and we decided to remove it from the text, in lines 100-101. Thanks.

8. *Line 146-147: It doesn't read well to say that 'municipalities' were 'conducted'.*

**Reply**: Changed to “analyzed” (line 133). Thanks.

9. *Lines 180-190: Could this section possibly be incorporated in the supplementary material?*

**Reply**: Yes, section was moved to Supplementary Material S2 (sample characteristics after applying the inclusion and exclusion criteria). Please, see Lines 152-165.

10. *Lines 207-209: Could this section be moved to supplementary note 2 where further information is available relating to data projections in the table of supplementary note 2. Currently, the table in supplementary note 2 is difficult to understand as it on its own with no further narrative.*

**Reply**: This explanation was replicated in Supplementary Material 2. In addition, the section has been expanded for further explanation. Please, see Lines 183-187 in the manuscript and Supplementary Material 2.

11. *Line 234: I think the word 'waves' or 'periods' would be better than 'panels'*

**Reply**: Changed to “periods” (line 199). Thanks.

12. *Table 2: The GDP per capita units are not clear. Is it measured in thousands or tens of thousands Brazilian reais?*

**Reply**: The municipal GDP was used at constant prices – BRL (thousands) making it possible to assess real municipal growth, based on an implicit deflator (index that measures the variation in price levels between the base year and the current year). The explanation was inserted in Tables 1 and 2. Thanks.

13. *Table 3: Northeast subtotal % formatting issue. Dash rather than a period for the decimal place. Make sure you consistently use a decimal point symbol. For the Southeast subtotal % 2010-2015 you use a comma instead of a period symbol. Perhaps double check to ensure formatting consistency.*

**Reply**: Changed to point: 46.0 (Northeast); 4.1 (Piaui); 22.3 (Southeast); 13.6 (Midwest). Thanks.

14. *Lines 328-350: Much of this is repeating the information that is in the supplementary section. I believe it would be better to focus on the model you are using and how to interpret it. You could also briefly mention that criteria were used for choosing this model based on its goodness of fit but save the detail on model selection and sensitivity for the supplementary sections.*

**Reply**: This section has been modified for clearer and more direct reading (see lines 296-302).

15. *Line 364: It would be helpful for the reader to consider what an increase of 1 in a logged explanatory variable means. Would it be more helpful to interpret the population coefficient by stating how this relates to percentage changes when measured in logs?*

**Reply**: The authors are grateful for the new observation. We clarify that the option of using a logarithmic scale in the population size variable is intended to just maintain a scale with less variability, since the variable presents high absolute values and it would difficult comparison of smaller cities with larger cities without any adjustment.

The general interpretation of this variable, based on results of the statistical model, has been added to lines 312-319 in order to analyze its overall effect. To this end, the main interpretation consists of noticing the direction (increase or decrease) of the variable's influence on prevalence. Thus, an increase of one unit in the natural logarithm scale (which corresponds to an increase of approximately 2.718 times in the original variable) would provide a decrease of 13.8% in the mean prevalence of the disease. Explanation added in Lines 319-320.

16. *Line 372: You state that 'based on these results' the decreasing prevalence between 1977 and 2013 was estimated. It is unclear how you determined 84.2% based on these results. Could you clarify?*

**Reply**: The results presented in Table 4 for the categorical variable representing the survey periods (Year), were estimated taking 1950 period as a reference. The interpretation of the coeficients estimates, as well as the relative ratio (RR) for periods of 1977 and 2013, are related to the prevalence situation of 1950 period. We chose to withdraw this estimate and explain better this result in lines 324-333. In addition, see comment 17 detailed.

17. *Lines 384-394: It would be helpful to explain more clearly why the model estimates an increases in prevalence between 1950-1977 while the descriptive statistics indicate a decrease. Why does prevalence increase (between these two points) when the other variables in the model are held constant? You state that this could be the result of the sampling procedures used which suggests that this may be confounding your results. This then raises the question as to whether the sampling procedure was sound to begin with.*

**Reply**: First, it should be noted that the objective of the research is to infer which explanatory variables may have significantly interfered in the reduction in the prevalence of *Schistosomiasis Mansoni* between the periods of 1947–1953, 1975–1979 and 2010–2015.

As the data collection periods are different, it is necessary to include a variable in the model that takes this fact into account, mainly because there is a very relevant time difference between the 3 surveys: more than 20 years (2 decades) between the 1st. sampling (1947-1953) and the 2nd. (1975–1979); over 60 years (6 decades) between 1st and 3rd (2010–2015); and over 40 years (4 decades) between 2nd and 3rd.

It is natural to expect that explanatory variables of a socioeconomic, population, education, basic sanitation, health, etc. changed over time between data collections. Under this aspect the analysis of the RR (Ratio Rate) for YEAR by itself is questionable, since the calculation of the RR presupposes the comparison of municipalities that would have the same values in all the explanatory variables that are part of the model, the only difference being the period (YEAR) of data collection.

Due to the nature of the explanatory variables included in the model (see Table 4 of the main manuscript) this hypothetical situation it is not expected mainly because the survey periods involve decades of distance.

It is true that when analyzing the descriptive results presented in Table 2 for observed prevalence of *Schistosomiasis* *mansoni,* there is an indication that the prevalence average of year 1977 is smaller than for year 1950 (4.8 and 8.3, respectively) while the RR value related to 1977 compared to 1950 suggests the opposite (RR=1.352). This is an example of what is called as Simpson's Paradox which is common when data is originally clustered into separate groups but clustering is ignored in the statistical analysis. The average is a simple view of the data, as it does not take into account grouping factors which are essential in the statistical analysis of *Schistosomiasis mansoni* prevalence due to the fact that in Brazil, region and states have specific social and economic characteristics besides geographical and climate differences. By not taking grouping into account, it is possible to see a different trend when looking at the results from a descriptive analysis and the results obtained from adjusting a multilevel model as presented in the paper (Table 4), since the adjusted model takes into consideration region and states as well as the fact that the municipalities of the three periods are not all the same and include effect of other covariates.

In order to better substantiate the solution presented in Table 4 (of the main manuscript), the estimate of the percentage of *Schistosomiasis mansoni* for each municipality in each sampling period was calculated using the values predicted by the statistical model presented in Table 4. The results of the descriptive analysis of the values obtained for each sampling period (variable called YEAR) are presented below (Auxiliary Table). This table was also included in the Supplementary Material 3 (Table S3.2). It can be seen that predicted prevalences are consistent with observed prevalences. In addition, an analysis of the municipal prevalences estimated by the model provided similar and consistent results with those observed in the data, corroborating the adequacy of the adjusted model (see Table S3.2 of Supplementary File 3). The analysis of results involving variable YEAR was changed in the manuscript. (Results, in Lines 324-333, and Discussion, in Lines 351-355)

Auxiliar Table: Descriptive statistics on the prevalence of schistosomiasis per study period in the 1,721 sampled Brazilian municipalities: Observed and predicted values using the zero-inflated Poisson multilevel regression model.

SD: Standard Deviation. n=Number of sampled municipalities.

18. *Line 422: 'immediate host for fetching water' is unclear.*

Reply: In orther to clarify the section, we modified sentence in Lines 379-382. “Although schistosomiasis is not a waterborne disease, adequate water supply is expected to be positively associated with its control, in avoiding the need for individuals to have contact with surface water in order to fetch water for household supply”

19. *Line 538: Am I correct in thinking that the use of random effects allows for better precision in this context but does not lead to better estimation of the coefficient itself. The writing here implies that there is somehow better estimation of the coefficient itself too?*

**Reply**: In this study, municipalities, states and regions were used as random effects. Models with random effects assume that the effect of interest is not the same in all periods. The random effects allowed to control the variation between municipalities, from sources of uncontrolled variation in the study.

Thus, these models provide more accurate estimates of the estimated parameters, which makes the test more sensitive to capture significant differences. Random effects are commonly incorporated into prospective data models to consider heterogeneity between observational units, accommodate dependencies on repeated observations, and study the structure of variance and covariance that best accounts for dependence among measures. We added references models making the estimates more accurate.

Refferences

B.M. Bolker, M.E. Brooks, C.J. Clark, S.W. Geange, J.R. Poulsen, M.H.H. Stevens, J.S.S. White Generalized linear mixed models: a practical guide for ecology and evolution Trends Ecol. Evol., 24 (2009), pp. 127-135

B.M.Bolker. Linear and Generalized Linear Mixed Models. In G.A.Fox,S.Negrete-Yankelevich,and V.J.Sosa,editors, Ecological Statistics. OxfordUniversityPress,Oxford,UK, 2015. [p378,379

20. *Line 572: I do not think it's correct to call this an 'international' study when it is focused on Brazil.*

**Reply**: Changed to “To the best of our knowledge, this is the first study that used a longitudinal epidemiological design to analyze data from national prevalence surveys covering a large period of many decades.” (lines 533-537)

21. *Supplementary Note S1: You refer to a power analysis. I believe you are explaining the power analysis conducted by previous authors rather than a power analysis carried out by you? Could you include a reference to the previous authors in this paragraph if this is the case?*

**Reply**: We clarify that the analysis for determining the sample was performed by National Survey on the Prevalence of Schistosomiasis and Soil-transmitted Helminth Infections (INPEG) (2010–2015) professionals. The authors did not participate in this analysis, so we added the reference in Supplementary Material S1. All information contained in that section refers to INPEG. Thanks.

22. *Supplementary Note S2, at the beginning of page 2: you mention that there were no significant differences between groups. It is not clear what variable is being tested.*

**Reply**: Thanks for this contribution. We clarify that the tests carried out confirmed that there was no difference between variable prevalence in the sample surveyed by the 1947-1953 survey when comparing to the final sample obtained after applying the inclusion and exclusion criteria (Group 1: considering sample of all municipalities were surveyed; Group 2: considering sample after applying the inclusion and exclusion criteria of the study). This information were inserted in Supplementary Note S2.

23. *Supplementary Note S2, Table on page 2: For consistency with the rest of the manuscript, use the period symbol for decimal places.*

**Reply**: changed to “33.33” and “100.00”. Thanks.

24. *Supplementary Note S2, Table on page 2: I think this table needs more explanation. For the first period you state the n=5442. But for the two variables with projections, there are 907 observations. So it seems like the 5442 is 907*6. However, this is a lot of work for the reader! Given that so many of the key explanatory variables are constructed using projections, I think the projection approach needs more careful discussion than is currently in the manuscript.*

**Reply**: Thanks for this contribution. We clarify that a descriptive and informative text was added to Table 1 of the supplementary material with details on the estimation techniques used, the variables, number of observations and justification for their use for each of the periods. References were also added to support the description.

We hope to have addressed all reviewers' concerns appropriately and respectfully, and we reiterate our thanks to the reviewers for their unvaluable assessment. We remain available for any further clarification.

Belo Horizonte, Brazil, May 07, 2023

Mariana Cristina Silva Santos

Guilherme Lopes de Oliveira

Sueli Aparecida Mingoti

Léo Heller
